# Energetic Materials Based on *N*-substituted 4(5)-nitro-1,2,3-triazoles

**DOI:** 10.3390/ma15031119

**Published:** 2022-01-31

**Authors:** Gennady T. Sukhanov, Yulia V. Filippova, Yuri V. Gatilov, Anna G. Sukhanova, Irina A. Krupnova, Konstantin K. Bosov, Ekaterina V. Pivovarova, Vyacheslav I. Krasnov

**Affiliations:** 1 Laboratory for Chemistry and Technology of High-Energy Azoles, Institute for Problems of Chemical and Energetic Technologies, Siberian Branch of the Russian Academy of Sciences (IPCET SB RAS), 659322 Biysk, Russia; suhanovlab7@mail.ru (G.T.S.); nika7_anna@mail.ru (A.G.S.); irinka-krupnova@mail.ru (I.A.K.); kosmos070@gmail.com (K.K.B.); pivovarova.ekaterina@inbox.ru (E.V.P.); 2 Department of Chemistry, Novosibirsk Institute of Organic Chemistry, Siberian Branch of the Russian Academy of Sciences, 630090 Novosibirsk, Russia; gatilov@nioch.nsc.ru (Y.V.G.); krasnov@nioch.nsc.ru (V.I.K.)

**Keywords:** *N*-substituted nitro-1,2,3-triazoles, high-energy ionic materials, regioselectivity, quaternization, complexation

## Abstract

The regularities and synthetic potentialities of the alkylation of 4(5)-nitro-1,2,3-triazole in basic media were explored, and new energetic ionic and nitrotriazole-based coordination compounds were synthesized in this study. The reaction had a general nature and ended with the formation of *N*1-, *N*2-, and *N*3-alkylation products, regardless of the conditions and reagent nature (alkyl- or aryl halides, alkyl nitrates, dialkyl sulfates). This reaction offers broad opportunities for expanding the variability of substituents on the nitrotriazole ring in the series of primary and secondary aliphatic, alicyclic, and aromatic substituents, which is undoubtedly crucial for solving the problems related to both high-energy materials development and medicinal chemistry when searching for new efficient bioactive compounds. An efficient methodology for the separation of regioisomeric *N*-alkyl(aryl)nitrotriazoles has been devised and relies on the difference in their basicity and reactivity during quaternization and complexation reactions. Based on the inaccessible *N*3-substitution products that exhibit a combination of properties of practical importance, a series of energy-rich ionic systems and coordination compounds were synthesized that are gaining ever-increasing interest for the chemistry of energy-efficient materials, coordination chemistry, and chemistry of ionic liquids.

## 1. Introduction

**Figure 1:**
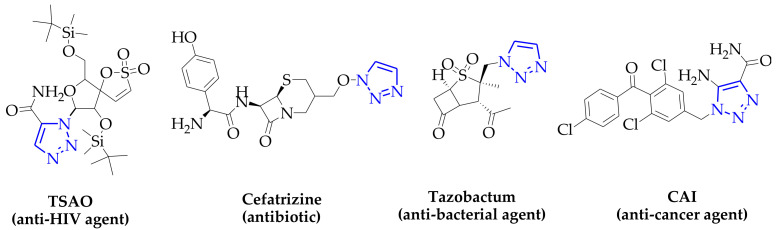
Some of the commercially available 1,2,3-triazole ring-based drugs.

1,2,3-Triazole derivatives are viewed as the valuable building block for the molecular construction of a wide array of new compounds with various properties of practical importance. The 1,2,3-triazole ring is a major pharmacophore system among nitrogen-containing heterocycles [1,2,3]. Furthermore, a number of drugs that contain 1,2,3-triazole moieties, including TSAO [4] (anti-HIV agent), Cefatrizine [5] (antibiotic), CAI [6] (anticancer agent), and Tazobactum [7] (antibacterial agent) are currently used in clinical applications (Figure 1).

The favorable properties of the enhanced biological activities of the triazole ring include hydrogen bonding capability under in vivo conditions, a strong dipole moment, high chemical stability (they are typically inert toward oxidizing and reducing agents), and rigidity [8].

1,2,3-Triazole nitro derivatives―five-membered heteroaromatic systems bearing three endocyclic nitrogen atoms and exocyclic explosophoric NO_2_ groups―are commonly used in the development of efficient high-energy compounds, including ionic ones, that exhibit enhanced technological and operational safety for various applications [9,10,11,12,13,14].

The aromatic nature provides the triazole heterocyclic molecule with high thermal and chemical stabilities and a low sensitivity to mechanical stimuli [9,10]. The three coupled nitrogen atoms united into the five-membered heterocyclic system preserve the energy potential of the azido group and impart quite a high enthalpy of formation to 1,2,3-triazoles [15]. The functionalization with supplemental energy-rich moieties in the form of NO_2_ groups promotes enhanced density and increases the number of oxidizing elements (oxygen balance) required to oxidize components and maximize the energetic potential of the overall system [16]. The capability of accepting various metal ions and oxoacid anions opens the door to the synthesis of various supramolecular ionic systems, including the oxygen-enriched ones in the active form, starting from cations of triazolium heterocycles [10]. Such ionic systems holds promise as energetic compounds, which concurrently combine low sensitivity to mechanical stimuli and high energetic performance [17].

The high potential for practical application of 1,2,3-triazole derivatives provides for the highly relevant problem of finding directed synthesis methods for various functionalized triazoles-bearing systems.

There are two well-known, conceptually distinct approaches for the synthesis of *N*-substituted 4-nitro-1,2,3-triazoles. The first approach refers to the heterocyclization of nitrogen derivatives that contain activated C–C, N–N, and C–N bonds. A good deal of original papers and review articles report the findings of the first approach [18,19]. However, such methods are limited by the synthesis directions of inaccessible *N*3-substituted nitro derivatives of 1,2,3-triazoles.

The rational method for the functionalization of 1,2,3-triazole nitro derivatives for the potential synthesis of *N*1, *N2,* and *N*3 isomers is by alkylating unsubstituted 4-nitro-1,2,3-triazoles. Varying the nature of substituents and their location within the structure of *N*-substituted nitrotriazoles imparts a specified set of characteristics to the compounds and allows the control of their biological activity, energy performance, complexing, and other useful properties.

The alkylation reaction of azoles is usually carried out in the presence of bases. In these reactions, nitrotriazoles manifest properties of ambident nucleophiles. The data available in the literature on the alkylation of 4-nitro-1,2,3-triazole (**1**) are limited, have no systematic nature, and tend to be controversial. Most of the examples described previously regarding the alkylation of triazole **1** in basic media highlight the formation of a mixture of only two isomers [20,21,22,23,24,25]. The reaction [20,21,22,23,24,25] between triazole **1** and different electrophilic agents in basic media ends with the resulting mixed reaction products related to *N*1- and *N*2-isomeric 4-nitro-1,2,3-triazoles. The reaction [24,25] between triazole **1** with propargyl bromide [24] and ethyl iodides [25] also shows the formation of only two isomers, to one of which the structure of *N*3-isomer (1-substituted 5-nitro-1,2,3-triazole) has been ascribed by mistake.

Along with that, inaccessible *N*3-substitution products in the series of isomeric *N*-substituted 4-nitro-1,2,3-triazoles are of special interest for the development of high-energy [26,27], ionic [28,29,30], and polymeric materials [31] and metal complexes [32,33]. Due to the low accessibility, N3-substitution products are almost understudied. At the same time, they may serve as promising cages for high-energy materials because they have a unique combination of extreme and practically important properties (high enthalpy of formation [34], basicity [35]). From the synthetic standpoint, *N*3 derivatives arouse a huge interest, as they are more efficient in quaternization [36] and complexation [32,33] reactions among the isomeric *N*-substituted 4-nitro-1,2,3-triazoles. Such reactions are a powerful tool to produce the important class of compounds—energy-rich ionic systems and coordination compounds starting from nitrotriazoles.

Thus, the present study was focused on the synthesis and transformations of a wide array of N-alkyl(aryl)-nitrotriazoles differing in substituent types. The present study reports the results of the alkylation of 4-nitro-1,2,3-triazole in alkali whereby the triazole heterocycle is functionalized over all the three endocyclic nitrogen atoms. The unique properties of the *N*3 isomers allow for new quaternization and complexation processes that afforded a series of ionic and coordination compounds that have a set of characteristics combining enhanced energy performance and safety.

## 2. Materials and Methods

All the reagents and solvents were used as received. Ethanol (EtOH) (96.2%) was obtained from Kirov BioChemPlant, Kirov, Russian Federation. Dimethyl sulfoxide-d6 (DMSO-d6) (99.9%), dimethyl sulfate (99.8%), and sodium carbonate (99.8%) were acquired from Chemical Line Co. Ltd., Saint Petersburg, Russian Federation. Dichloromethane (99.8%), MgSO_4_·7H_2_O (99.5%), sodium hydroxide (99.6%), potassium hydroxide (85.7%), perchloric acid (72.8%), CuCl2·2H2O (99.0%), diethyl ether (99.2%), and tert-butanol (99.4%) were obtained from Vekton, Saint Petersburg, Russian Federation. Diethyl sulfate (98.0%), iodomethane (99.0%), bromoethane (99.0%), 1-bromopropane (99.0%), 2-bromopropane (99.0%), 1-bromo-3-methylbutane (99.0%), benzyl chloride (99.0%), and 2-ethylhexyl nitrate (97.0%) were procured from Aldrich, St. Louis, MO, USA. 1-Bromobutane (99.0%) was obtained from Sigma-Aldrich, St. Louis, MO, USA. Triazole **1** was prepared by the common procedure [37].

^1^H and ^13^C NMR spectra were recorded on a Bruker Avance III spectrometer (Bruker Corporation, Billerica, MA, USA). 1H NMR spectra were acquired at 400.13 MHz, while ^13^C NMR spectra were taken at 100.61 MHz. The measurements were conducted at 298 K unless otherwise stated. The spectra were calibrated using residual solvent signals (DMSO-*d_6_*: 2.50 ppm for ^1^H, 39.5 ppm for ^13^C). All NMR spectra of the new compounds are shown in the Supplementary Materials (Figures S1–S21). IR spectra (KBr): Simex FT-801 FTIR spectrometer (Simex, Novosibirsk, Russia). The melting point was determined on a Stuart SMP30 apparatus (Bibby Scientific Ltd., Stone, Staffordshire, UK). Elemental analyses were done on a Thermo Scientific Flash EA1112 CHNS elemental analyzer (Thermo Fisher Scientific, Waltham, MA, USA) for carbon, hydrogen, nitrogen, and oxygen contents. The XRD analysis was performed on a Bruker KAPPA APEX II CCD diffractometer (Bruker AXS GmbH, Karlsruhe, Germany) (λ(MoKα) = 0.71073 Å, φ,ω-scans of narrow (0.5°) frames). The density of the synthesized samples was measured on a AccuPyc II 1340 helium pycnometer (Micromeritics, Norcross, GA, USA) at 25 °C.

The reagents were procured from commercial sources and used as received unless otherwise stated. The commercially available compounds were used without additional purification unless otherwise stated. Triazole **1** was synthesized by the procedure reported [37].

**Synthesis of triazoles 2–4a**, **2–4b**, **2–4c**, **2–4d**, **2–4e**, **2–4f**, **2–4g**, **2–4h (general procedure)**. A suspension of triazoles **1** (2.85 g, 25 mmol) in ethanol (15 mL) (or water, 7.5 mL) and the corresponding alkali metal hydroxide (25 mmol) was heated to 40 °C with stirring until a solution was prepared. Then, dialkyl sulfate (benzyl chloride) (22.5 mmol, 0.9 equiv) or alkyl halide (50 mmol) was added and stirred. Low-boiling alkyl halides (EtBr, Pr^i^Br) were added dropwise with constant stirring. The reaction temperature and time for each case are summarized in Table 1. After the reaction was completed, the reaction mixture in ethanol was cooled to room temperature and concentrated in a rotary evaporator. The residue was treated with CH_2_Cl_2_ (3 × 25 mL). The reaction mixture in water was cooled, and extraction with CH_2_Cl_2_ (3 × 25 mL) was performed. The combined organic layers were washed successively with 3% aqueous sodium carbonate (7.5 mL), water (7.5 mL), dried over MgSO_4_, and then concentrated in a rotary evaporator. The overall yield and the composition of mixed alkylation products are listed in Table 1.

**Synthesis of triazoles 2–4j**. To a suspension of triazoles **1** (2.85 g, 25 mmol) in ethanol (15 mL) was added an equimolar quantity of sodium hydroxide and cyclohexyl nitrate (22.5 mmol) and stirred at 78–80 °C for 13 h. ^1^H NMR spectroscopy identified the formation of three isomeric N-cyclohexylnitrotriazoles **2–4j** (conversion degree of the starting cyclohexyl nitrate did not exceed 1%) in the reaction mixture. The reaction mixture was cooled to room temperature and ethanol was removed in vacuo. To the residue was added water (7.5 mL), the whole mixture was heated to 90–95 °C and held for 25 h with stirring. The product was isolated in a manner similar to the previous procedure. The overall yield and the ration of isomers **2–4j** are specified in Table 1.

**General procedure for quaternization of mixed 2–4a, 2–4b, 2–4c, 2–4d, 2–4e, 2–4f, 2–4g, 2–4h or 2–4j in t-BuOH-HClO_4_ (synthesis of 1-tert-butyl-3-alkyl-4-nitro-1,2,3-triazolium salts 5a–j)**.

A solution of the corresponding mixed triazoles **2–4a**, **2–4b**, **2–4c**, **2–4d**, **2–4e**, **2–4f**, **2–4g**, **2–4h** or **2–4j** (20 mmol) and *tert*-butanol (20 mmol) in conc. HClO_4_ (72%, 3.8 mL) was stirred for 12 h, and then, water was added (4 mL). The precipitated product was combined by filtration, washed with water, and dried to yield 1-*tert*-butyl-3-alkyl-4-nitro-1,2,3-triazolium salts **5a–d**, **5g**, **5j**.

Salts **5e**,**f**,**h** were isolated as follows: after being diluted, the reaction mixture was extracted with CH_2_Cl_2_ (20 mL) to recover products **5e**,**f**,**h** together with unreacted triazoles **2e**,**f**,**h** and **3e**,**f**,**h**. The organic layer was concentrated in a rotary evaporator and the residue was treated with Et_2_O. The precipitated products were combined by filtration and washed with Et_2_O to furnish salts **5e**,**f**,**h**.

**Scheme 1:**
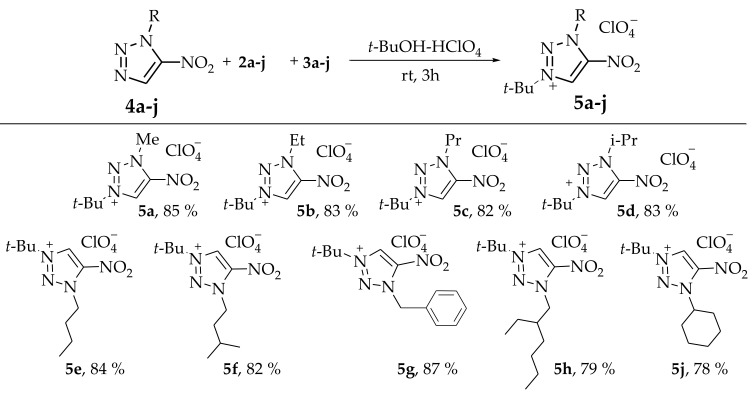
Regioselective quaternization of N-alkyl-4-nitro-1,2,3-triazoles in t-BuOH-HClO_4_.

The yields of salts **5a-j** were calculated equivalent to the proportions of *N*3 isomers **4a–j** in mixtures **2–4a, 2–4b**, **2–4c**, **2–4d**, **2–4e**, **2–4f**, **2–4g**, **2–4h**, or **2–4j** (Table 1, Scheme 1).

Salts **5a–d** and **5j** were compatible by the spectral characteristics with the compounds synthesized and characterized previously [36].

**1-Tert-butyl-3-n-butyl-4-nitro-1,2,3-triazolium perchlorate** (**5e**)


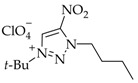


White solid. Yield: 84%, 0.33 g. Mp: 164–165 °C (H_2_O). ^1^H NMR (400 MHz, DMSO-d_6_): δ 0.95 (t, 3H, *J* = 7.4 Hz, C-CH_3_), 1.37–1.46 (m, 2H, C-CH_2_), 1.75 (s, 9H, C(CH_3_)), 1.95–2.02 (m, 2H, C-CH_2_), 4.93 (t, 2H, *J* = 7.2 Hz, N-CH_2_), 10.40 (s, 1H, =C-H) ppm. ^13^C NMR (100 MHz, DMSO-d_6_): δ 13.3 (CH_3_), 18.8 (CH_2_), 28.2 ((CH_3_)_3_), 29.5 (CH_2_), 55.0 (N-CH_2_), 68.7 (N-C), 128.6 (=C-H), 145.1 (C-NO_2_) ppm. FTIR (KBr): 624, 747, 844, 1030, 1092, 1191, 1334, 1366, 1381, 1469, 1546, 1582, 2944, 2977, 3096 cm^−^^1^. Elemental analysis: calcd. C 36.76, H 5.86, N 17.15, O 29.38; found C 36.68, H 5.74, N 17.08, O 29.12.

**1-Tert-butyl-3-i-amyl-4-nitro-1,2,3-triazolium perchlorate** (**5f**)


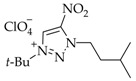


White solid. Yield: 82%, 0.34 g. Mp: 152–153 °C (H_2_O). ^1^H NMR (400 MHz, DMSO-d_6_): δ 0.97 (d, 3H, *J* = 6.6 Hz, C-(CH_3_)_2_), 1.67–1.75 (m, 1H, C-CH), 1.75 (s, 9H, C(CH_3_)), 1.88–1.93 (m, 2H, C-CH_2_), 4.95 (t, 2H, *J* = 7.6 Hz, N-CH_2_), 10.40 (s, 1H, =C-H) ppm. ^13^C NMR (100 MHz, DMSO-d_6_): δ 22.4 ((CH_3_)_2_), 25.4 (CH), 28.6 ((CH_3_)_3_), 31.2 (CH_2_), 54.2 (N-CH_2_), 69.2 (N-C), 129.1 (=C-H), 145.5 (C-NO_2_) ppm. FTIR (KBr): 624, 747, 845, 1093, 1191, 1162, 1380, 1467, 1547, 1584, 2937, 2970, 3103 cm^–^^1^. Elemental analysis: calcd. C 38.77, H 6.21, N 16.44, O 28.17; found C 38.70, H 6.14, N 16.38, O 28.14.

**1-Tert-butyl-3-benzyl-4-nitro-1,2,3-triazolium perchlorate** (**5g**)


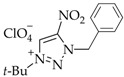


White solid. Yield: 87%, 0.38 g. Mp: 137–139 °C (H_2_O). ^1^H NMR (400 MHz, DMSO-d_6_): δ 1.71 (s, 9H, C(CH_3_)), 6.17 (s, 2H, N-CH_2_), 7.43–7.49 (m, 5H, CH_arom_), 10.43 (s, 1H, =C-H) ppm. ^13^CNMR (100 MHz, DMSO-d_6_): δ 28.4 ((CH_3_)_3_), 58.3 (N-CH_2_), 69.3 (N-C), 129.2–129.5 (CH_arom_), 129.6 (=C-H), 131.0 (C_arom_), 145.0 (C-NO_2_) ppm. FTIR (KBr): 626, 693, 717, 830, 855, 1028, 1087, 1190, 1259, 1297, 1342, 1379, 1457, 1544, 1583, 2995, 3100 cm^−^^1^. Elemental analysis: calcd. C 43.28, H 4.75, N 15.53, O 26.61; found C 43.12, H 4.68, N 15.48, O 26.57.

**1-Tert-butyl-3-ethylhexyl-4-nitro-1,2,3-triazolium perchlorate** (**5h**)


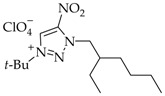


White solid. Yield: 79%. Mp: 149–150 °C (H_2_O). ^1^H NMR (400 MHz, DMSO-d_6_): *δ* 0.87–0.93 (m, 6H, 2CH_3_), 1.24–1.41 (m, 8H, CH(CH_2_)CH_2_CH_2_CH_2_),1.75 (s, 9H, C(CH_3_)), 2.09–2.15 (m, 1H, CH(CH_2_)CH_2_CH_2_CH_2_), 4.84 (d, 2H, *J* = 7.0 Hz, N-CH_2_), 10.42 (s, 1H, =C-H) ppm. ^13^C NMR (100 MHz, DMSO-d_6_): δ 10.4 (CH_3_), 14.4 (CH_3_), 22.8 (CH_2_), 23.1(CH_2_), 27.9 (CH_2_), 28.6 ((CH_3_)_3_), 29.6 (CH_2_), 38.1 (CH), 58.3 (N-CH_2_), 69.2 (N-C), 129.4 (=C-H), 145.6 (C-NO_2_) ppm. FTIR (KBr): 625, 745, 845, 1095, 1164, 1190, 1379, 1466, 1548, 1587, 2860, 2934, 2961, 3133 cm^−^^1^. Elemental analysis: calcd. C 43.92, H 7.11, N 14.63, O 25.07; found C 43.88, H 7.07, N 14.72, O 25.12.

After *N*3-isomers **4a–j** were isolated, the mixtures composed of *N*1-(**2a–j**) and *N*2-substituted derivatives (**3a–j**) were separated as follows: readily volatile isomers **3** were removed from mixed liquid compounds **2**,**3b–f**, **2**,**3h**, and **2**,**3j** by vacuum distillation and from mixed crystalline compounds **2**,**3a** and **2**,**3g** by extraction with nonpolar solvents. The residue was crystallized to yield individual *N*1 isomers **2a–j**. The compounds N-methyl-(**2–4a**), N-ethyl-(**2–4b**), N-*n*-propyl-(**2–4c**), N-isopropyl- (**2–4d**)**,** N-cyclohexylnitrotriazoles (**2–4j**), 1-*n*-butyl-4-nitro- (**2e**), 1-*n*-butyl-5-nitro-1,2,3-triazole (**4e**), 1-*i*-amyl-4-nitrotriazole (**2f**), and 1-benzyl-4-nitrotriazole (**2g**) corresponded by spectral characteristics to the compounds synthesized and characterized in the previous studies [20,32,35,38,39,40].

**2-n-Butyl-4-nitro-1,2,3-triazole** (**3e**)


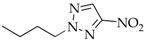


Colorless liquid. Yield: 39%, 1.7 g. Bp: 98–99 °C/1–2 mmHg). ^1^H NMR (400 MHz, DMSO-d_6_): δ 0.84 (t, 3H, *J* = 7.4 Hz, C-CH_3_), 1.22–1.31 (m, 2H, C-CH_2_), 1.87–1.95 (m, 2H, C-CH_2_), 4.52 (t, 2H, *J* = 7.1 Hz, N-CH_2_), 8.33 (s, 1H, =C-H) ppm. ^13^C NMR (100 MHz, DMSO-d_6_): δ 12.7 (CH_3_), 19.1 (CH_2_), 30.8 (CH_2_), 56.0 (N-CH_2_), 130.5 (=C-H), 152.9 (C-NO_2_) ppm. FTIR (KBr): 674, 758, 827, 862, 1025, 1103, 1296, 1340, 1444, 1537, 2876, 2962, 3145 cm^−^^1^. Elemental analysis: calcd. C 42.35, H 5.92, N 32.92, O 18.80; found C 42.40, H 5.90, N 32.97, O 18.74. 

**2-i-Amyl-5-nitro-1,2,3-triazole** (**3f**)


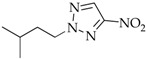


Colorless liquid. Yield 39%, 1.8 g. Bp 95–96 °C/1–2 mmHg). ^1^HNMR (400 MHz, DMSO-d_6_): δ 0.88 (d, 6H, *J* = 7.0 Hz, C-(CH_3_)_2_), 1.48–1.56 (m, 1H, C-CH), 1.81–1.87 (m, 2H, C-CH_2_), 4.54 (t, 2H, *J* = 7.3 Hz, N-CH_2_), 8.28 (s, 1H, =C-H) ppm. ^13^C-NMR (100 MHz, DMSO-d_6_): δ 21.4 ((CH_3_)_2_), 25.0 (CH), 37.5 (CH_2_), 54.6 (N-CH_2_), 130.3 (=C-H), 152.9 (C-NO_2_) ppm. FTIR (KBr): 675, 758, 826, 859, 1024, 1110, 1296, 1342, 1446, 1538, 2872, 2960, 3150 cm^−^^1^. Elemental analysis: calcd. C 45.64, H 6.57, N 30.42, O 17.37; found C 45.57, H 6.45, N 30.38, O 17.42.

**2-Benzyl-4-nitro-1,2,3-triazole** (**3g**)


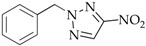


White solid. Yield: 25%, 1.1 g. Mp: 68–70 °C (EtOH). ^1^H-NMR (400 MHz, DMSO-d_6_): δ 5.81 (s, 2H, N-CH_2_), 7.35–7.40 (m, 5H, CH_arom_), 8.74 (s, 1H, =C-H) ppm. ^13^C-NMR (100 MHz, DMSO-d_6_): δ 59.4 (N-CH_2_), 128.5–128.8 (CH_arom_), 131.9 (=C-H), 134.1 (C_arom_), 153.3 (C-NO_2_) ppm. FTIR (KBr): 3139, 1952, 1774, 1525, 1442, 1383, 1354, 1325, 1309, 1171, 1074, 1028, 930, 891, 829, 760, 710, 691, 673 cm^−^^1^. Elemental analysis: calcd. C 52.94, H 3.95, N 27.44, O 15.67; found C 53.01, H 3.89, N 27.40, O 15.72.

**2-Ethylhexyl-4-nitro-1,2,3-triazole** (**3h**)


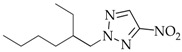


Colorless liquid. Yield: 33%, 1.8 g. Bp: 119–120 °C/−2 mmHg). ^1^H NMR (400 MHz, DMSO-d_6_): δ 0.81–0.87 (m, 6H, 2CH_3_), 1.22–1.28 (m, 8H, CH(CH_2_)CH_2_CH_2_CH_2_), 1.99 (m, 1H, CH(CH_2_)CH_2_CH_2_CH_2_), 4.46 (d, 2H, *J* = 6.7 Hz, N-CH_2_), 8.53 (s, 1H, =C-H) ppm. ^13^C NMR (100 MHz, DMSO-d_6_): δ 9.8 (CH_3_), 13.3 (CH_3_), 22.4 (CH_3_), 23.2 (CH_2_), 27.9 (CH_2_), 29.8 (CH_2_), 39.6 (CH), 59.1 (N-CH_2_), 130.4 (=C-H), 152.9 (C-NO_2_) ppm. FTIR (KBr): 677, 758, 827, 1026, 1180, 1295, 1341, 1385, 1446, 1541, 2931, 2960 cm^−^^1^. Elemental analysis: calcd. C 53.08, H 8.02, N 24.76, O 14.14; found C 53.11, H 8.07, N 24.81, O 14.23.

**2-Cyclohexyl-4-nitro-1,2,3-triazole** (**3j**)


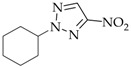


White solid. Yield 3.5%, 0.15 g. Mp 43–45 °C (EtOH). ^1^H-NMR (400 MHz, DMSO-d_6_): δ 1.20–1.31 (m, 1H, 4′-CH_2_), 1.40–1.50 (m, 2H, 3′,5′-CH_2_), 1.63–1.67 (m, 1H, 4′-CH_2_), 1.75–1.85 (m, 4H, 2′,3′,5′,6′-CH_2_), 2.14–2.17 (m, 2H, 2′,6′-CH_2_), 4.62–4.70 (m, 1H, N-CH), 8.71 (s, 1H, =C-H) ppm. ^13^C-NMR (100 MHz, DMSO-d_6_): δ 24.0 (2CH_2_), 24.5 (CH_2_), 31.8 (2CH_2_), 65.0 (N-CH), 131.0 (=C-H), 152.6 (C-NO_2_) ppm. FTIR (KBr): 696, 760, 833, 882, 986, 1034, 1351, 1405, 1446, 1482, 1536, 1766, 2854, 2928, 3140 cm^−1^. Elementary analysis: calcd. C 48.97, H 6.16, N 28.56, O 16.31; found C 48.86, H 6.22, N 28.61, O 16.32.

**Complex 6**. To a solution (3.12 g, 20.0 mmol) of mixed nitrotriazoles **2**–**4c** (ratio of **2c**:**3c**:**4c** = 35:56:9) in 96% EtOH (1 mL) was added a solution (0.31 g, 1.8 mmol) of CuCl_2_·2H_2_O in EtOH (2 mL). The whole was stirred at room temperature and held for about 1 month to furnish crystals of complex Cu_4_OCl_6_L_4_, where L = 1-*n*-propyl-5-nitro-1,2,3-triazole **4c**.

Green crystals. Yield: 0.09 g (4.4%). ^1^H NMR (400 MHz, DMSO-d_6_): δ 0.85 (br s, 3H, N-CH_3_), 1.85 (br s, 2H, N-CH_2_), 4.60 (br s, 2H, N-CH_2_), 8.63 (br s, 1H, C-H) ppm. ^13^C NMR (100 MHz, DMSO-d_6_): δ9.4 (N-CH_3_), 20.8 (N-CH_2_), 51.4 (N-CH_2_), 132.1 (C-H), 142.9 (C-NO_2_) ppm. FTIR (KBr): 697, 745, 802, 835, 877, 997,1098, 1162, 1222, 1294, 1368, 1431, 1464, 1521, 1563, 2879, 2973, 3110, 3156 cm^−^^1^.

**X-ray Crystallography**

Single crystal X-ray diffraction intensity data were collected at 296(2) K using a Bruker APEX-II CCD diffractometer equipped with graphite monochromated MoKα radiation (λ = 0.71073 Å). Data reduction was carried out using the program Bruker SAINT, and an empirical absorption correction was applied with the Bruker SADABS program based on the multi-scan method. The structure of the complex was solved by the direct method (SHELXT-18) and refined by the full-matrix least-square technique (SHELXL-18) with anisotropic thermal parameters. All hydrogen atoms were refined isotropically in riding positions. CCDC 2119451 contains the supplementary crystallographic data of 6. These data can be obtained free of charge from the Cambridge Crystallographic Data Centre.

More information can be found in the Supplementary Materials (Tables S1 and S2).

## 3. Results and Discussion

The first phase of this study was to explore the alkylation regularities of triazole **1** in basic media. All the three available reaction sites―*N*1, *N2,* and *N*3 heteroatoms―were found to undergo an electrophilic attack, irrespective of the alkylating agent nature (alkyl- or aryl halides, alkyl nitrates, dialkyl sulfates), solvent and base types, and reaction temperature and time. All the cases resulted in high yields of three regioisomeric *N*-alkylation products **2–4** among which *N*2-substituted derivatives **3** were mostly prevailing (Table 1).

At the first stage, the equimolar quantity of alkali produced easily a highly nucleophilic anion from *NH*-triazole **1** that had quite a high acidity (pKa 4.8). The latter underwent an attack by different electrophilic agents to furnish *N*-substitution products.

The structure of the alkylating reagent determined significantly the reaction conditions and the ratio of alkylation products. The reaction with dialkyl sulfates (DAS) at 78 °C in ethanol or at 84–90 °C in water in excess of triazoles **1** was completed within 5–10 min (Entries 1–4). Prolongation of the reaction involving *N*-substituted azoles, including *N*-alkyl-4-nitro-1,2,3-triazoles [20,38], in acidic media or by using activated alkylating reagents may be accompanied by the migration of substituents and interconversion of regioisomers. An increase in time of the reaction between triazole **1** and diethyl sulfate from 5 min to 10–15 h did not considerably alter the composition and ratio of isomers **2–4b** (Entries 4–6).

To exclude possible quaternization processes when triazole **1** is reacted with DAS, the alkylating reagent was used in deficiency (0.9 equiv). Since alkyl halides do not engage in the quaternization reaction with *N*-substituted 4-nitro-1,2,3-triazole, excess alkyl halides (2–3 equiv) were utilized in the alkylation of triazole **1**, which is especially important when using lower alkyl halides because of possible reaction losses associated with their low boiling points. When alkyl halides or DAS were employed in ethanol, isomer **3** was prevailing in mixed alkylation products **2–4** in all cases. The proportion of **3** in the mixture was 53–57% (Entries 1, 3, 4, 7–13). The alkylation conditions using water as the polar solvent increased naturally a proportion of the most polar *N*1-isomer **2**, in which case one of regioisomers **2** or **3** (Entries 2, 4–6, 9) was observed to dominate slightly (Entries 2, 4–6, 9).

The content of minor *N*3-isomer **4** in the mixed alkylation products of triazole **1** and dialkyl sulfates was 11–12% (Entries 1–4) and did not exceed 9% when alkyl halides were used (Entries 7–16). A minimum proportion of isomer **4** (as little as 2%, Entry 15) was documented in the reaction with ethylhexyl nitrate, which is likely due to the steric effects of the ethylhexyl substituent. Unexpectedly, when cyclohexyl nitrate was used, the composition of the isomers changed dramatically, and the proportion of **4** came up to 18% (Entry 16). Moreover, the alkylation reaction between triazoles **1** and cyclohexyl nitrate in basic media considerably diminished the yield of products **2–4j**. The yield of the target *N*-cyclohexylnitrotriazoles **2–4j** in water was not above 8%, and when reacted in alcohol, the products were documented only in the ^1^H NMR spectra. This is attributed to the tendency of cyclohexyl nitrate to undergo a side elimination reaction to form cyclohexene. The latter did not probably participate in the primary reaction; instead, it was consumed during polymerization. As opposed to the conditions considered, unsaturated alicycles have successfully been used to alkylate azoles in acidic media [41,42].

Benzyl chloride used as the activated electrophilic reagent afforded mixed regioisomers containing chiefly *N*1-substitution product **2h**. The proportion of **2h** in the mixture reached 64% (Entry 14).

**Figure 2:**
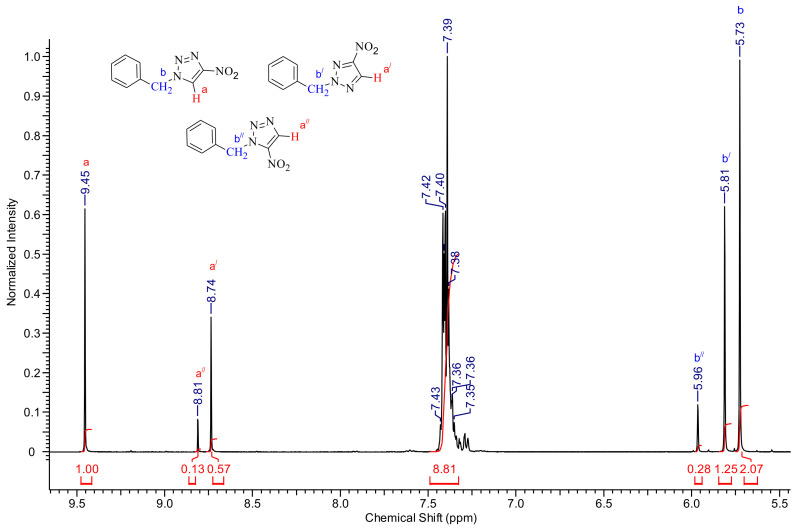
A characteristic ^1^H NMR spectrum of isomeric N-alkyl-4-nitro-1,2,3-triazoles by the example of triazoles **2–4g**.

The composition of the alkylation products and the ratio of isomeric *N*1-, *N*2- and *N*3-alkyl-4-nitro-1,2,3-triazoles **2–7a–c** were estimated from the integral intensities of characteristic proton signals of the cyclic carbon atom of the H-5 heterocycle in the ^1^H NMR spectra. For the signal assignment in the ^1^H NMR spectra, the known regularity for alkyl nitrotriazoles [32,35,38] was employed whereby the H-5 proton signals of the nitro-1,2,3-triazole ring are always arranged in the sequence: δ (H-5 *N*1-isomer) > δ (H-5 *N*3-isomer) > δ (H-5 *N*2-isomer). For example, such signals for compounds **2g** (*N*1-isomer), **3g** (*N*2-isomer), and **4g** (*N*3-isomer) are observed at 9.45, 8.74, and 8.81 ppm, respectively. In addition, the spectrum showed pronounced proton signals from the benzyl substituents: singlets of methyls associated with endocyclic nitrogen atoms were recorded at 5.73, 5.81, and 5.96 ppm for isomers **2g**, **3g**, and **4g**, respectively. The proton signals from the benzyl rings for isomers **2–4g** were close and observed near 7.27–7.43 ppm (Figure 2).

Regioisomerism exerts a significant impact on the physicochemical and energetic characteristics of *N*-alkyl(aryl)-4-nitro-1,2,3-triazoles **2a–j**, **3a–j**, **4a–j** (Table 2). It was found that *N*3-derivatives in the series of regioisomeric *N*-substituted 4(5)-nitro-1,2,3-triazoles have the highest density and enthalpy of formation, making them a promising scaffold for the construction of high-energy compounds.

The basicity of *N*-alkyl-4-nitro-1,2,3-triazoles as per quantum–chemical predictions is also dependent on the location of the substituent on endocyclic nitrogen atoms in the nitrotriazole ring and increases in the row: *N*2- <*N*1- <*N*3-isomer. The calculated *pK*_BH+_ value of 1-methyl-5-nitro-1,2,3-triazole (*N*3-isomer) is more than 4 log units higher than those of the *N*1-substituted derivatives [35].

The high basicity of the *N*3-isomers makes them attractive from the synthetic perspective. The dramatic difference of the *N*3 isomers from other regioisomers is that they are involved in complexation and quaternization reactions in acidic media. For instance, the use of efficient *t*-BuOH-HClO_4_ allows for the selective quaternization of *N*3-isomers **4a–j**. The quaternization of isomers **4a–j** proceeded over the *N*1 atom to furnish 1-(*tert*-butyl)-3-alkyl-4-nitro-1,2,3-triazolium perchlorates **5a–j** in a high yield of 78–87% (Scheme 1). The yield of salts **5a–j** was calculated in equivalent to the portion of *N*3-isomers **4a–j** in mixed **2–4a**, **2–4b**, **2–4c**, **2–4d**, **2–4e**, **2–4f**, **2–4g**, **2–4h** or **2–4j**.

Nitrotriazolium salts **5a–d**, **5g**, **5j** (where R = Me, Et, Pr, i-Pr, Bn, cyclohexyl) precipitated as crystals from the reaction mixture. As the length of the substituent (R = Bu, i-amyl, 2-ethylhexyl) increased due to the higher solubility, salts **5e**,**f**,**h** required other isolation conditions: products **5e**, **f**, and **h** were extracted with dichloromethane from the water-diluted reaction mixture; the solvent was removed in vacuo; they were treated with diethyl ether, and the precipitated products were combined by filtration to yield salts **5e**,**f**,**h** individually.

**Scheme 2:**
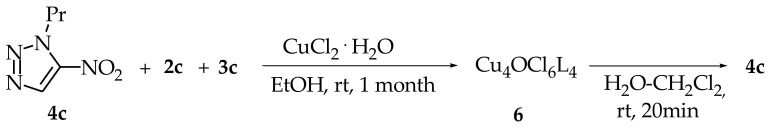
Complexation of triazole **4c**.

Treatment of mixed N-alkyl nitrotriazoles **2–4c** with copper(II) chloride afforded coordination compound **6** in which *N*3-isomer **4c** acted as the monodentant ligand (L) due to the *N*1 atom being involved in the coordination (Scheme 2). The selective formation of complex isomer **4c** was also due to the higher basicity of the *N*3 derivatives from among their regioisomers **2–4c [35]**. The resultant complex **6** was easily decomposed by water to form free ligand **4c**.

Thus, the difference in basicity [35] and reactivity during the quaternization [36] and complexation [32,33] processes typical of isomeric alkyl nitrotriazoles was used herein to isolate inaccessible *N*3-substituted derivatives from the resultant mixed regioisomers **2a–j**, **3a–j** and **4a–j** and use them for the synthesis of high-energy ionic materials and coordination compounds.

After *N*3 isomers **4a–j** were isolated, the mixtures composed of *N*1-(**2a–j**) and *N*2-substituted derivatives (**3a–j**) were separated as below. Highly volatile isomers **3** were removed from mixed liquid compounds **2, 3b–f, 2, 3h, 2**, and **3j** by vacuum distillation, and from mixed crystalline compounds **2, 3a, 2,** and **3g** by extraction with nonpolar solvents. The residue was crystallized to give individual *N*1-isomers **2a–j**.

**Figure 3:**
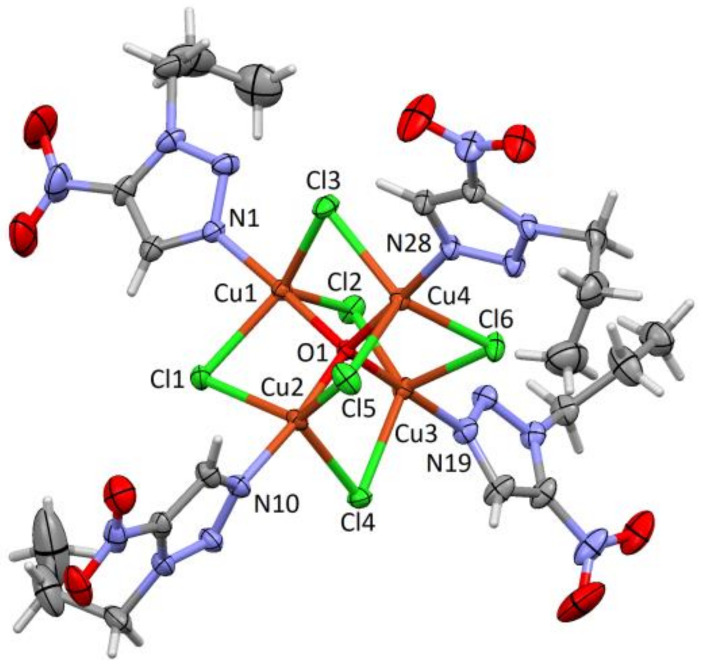
Structure of complex **6** with displacement ellipsoid at the 30% probability.

The structure of complex **6** (CCDC-2119451) [43] was unambiguously validated by single-crystal X-ray diffraction (Figure 3). The copper atoms of tetranuclear copper(II) complex **6** exist in a distorted trigonal-bipyramidal environment, with the N triazole atoms and the central oxygen atom in axial positions. Three chlorine atoms lie in the equatorial plane with the Cu atom deviations by 0.230, 0.186, 0.225, and 0.211 Å directed to the N atom accordingly for Cu1, Cu2, Cu3, and Cu4. The Cu–O, Cu–Cl, and Cu–N lengths range from 1.892(1), 2.3384(6), and 1.972(2) Å to 1.904(1), 2.5233(7), and 1.979(2) Å, respectively. For example, these ranges are close to the analogous ranges for hexakis(µ-chlorido)-(µ-oxido)-tetrakis(1-vinyl-1H-imidazole)-tetra-copper(II) [44] and (µ4-oxo)-hexakis(µ2-chloro)-tetrakis(1-ethyl-5-nitro-1,2,3-tetrazol-3-yl)-tetra-copper(II) [32]. Interestingly, the three propyl groups are oriented in gauche conformation, while the propyl of N19 triazole cycle is in anti-conformation. The shortest intermolecular contact is C-H...O with an H...O distance of 2.50 Å between the propyl and nitro groups leading to the formation of centrosymmetric dimers.

## 4. Conclusions

Synthetic methods for a wide array of *N*-alkyl(aryl)-4(5)-nitro-1,2,3-triazoles (alkyl = Me, Et, Pr, Pr^i^, Bu, 2-ethylhexyl, cyclohexyl; aryl = Bn), including inaccessible *N*3-substitution products, have been devised herein. Due to the unique physicochemical characteristics (the highest enthalpies of formation, density, basicity), the inaccessible *N*3-substitutuion products appeared to be quite attractive as cages for the construction of energetic ionic and coordination compounds. The synthesis involved the N-monoalkylation in basic media, quaternization using the highly efficient *t*-BuOH–HClO_4_ system, and complexation with transition metal salts. The methodology for the separation of regioisomeric *N*-alkyl(aryl)nitrotriazoles by quaternization and complexation reactions warranted the synthesis of a range of new energy-efficient nitrotriazole salts and coordination compounds.

## Figures and Tables

**Table 1:** Alkylation of 4(5)-nitro-1,2,3-triazole with different alkylating agents.

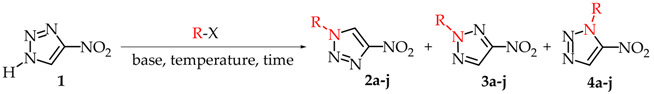								
Entry	R	X	2−4	Solvent	Temp, °C	Time, h	Yield, %	2:3:4 Ratio ^c^
1	Me	MeOSO_3_	a	EtOH	78	10 ^b^	83	36:53:11
2	Me	MeOSO_3_	a	H_2_O	84	5 ^b^	81	45:43:12
3	Et	EtOSO_3_	b	EtOH	78	10 ^b^	81	33:56:11
4	Et	EtOSO_3_	b	H_2_O	84	5 ^b^	85	40:48:12
5	Et	EtOSO_3_	b	H_2_O	90	10	82	42:47:11
6	Et	EtOSO_3_	b	H_2_O	90	15	80	41:48:11
7	Me	I	a	EtOH	28	2	79	40:56:4
8 ^a^	Et	Br	b	EtOH	70	10	85	37:57:6
9	Et	Br	b	H_2_O	50	25	85	43:50:7
10	n-Pr	Br	c	EtOH	70	10	80	35:56:9
11	i-Pr	Br	d	EtOH	68	10	82	35:56:9
12	n-Bu	Br	e	EtOH	80	5	82	41:53:6
13	i-amyl	Br	f	EtOH	80	5	82	41:53:6
14	Bn	Cl	g	EtOH	80	5	88	64:30:6
15	2-ethylhexyl	ONO_2_	h	EtOH	78–100	10	78	53:45:2
16	cyclohexyl	ONO_2_	j	EtOH-H_2_O	78–95	25	8	37:45:18

Note: ^a^ KOH was used as the base, while NaOH was used in all of the other reactions. ^b^ Time is expressed in minutes.^c^ Ratios obtained by comparing peak integrations in the ^1^H NMR spectrum of the crude reaction product.

**Table 2:** Density and enthalpy of formation of regioisomeric N-methyl-4(5)-nitro-1,2,3-triazoles **2–4a**.

	*N*1-Isomer 2a	*N*2-Isomer 3a	*N*3-Isomer 4a
ρ, g/cm^3^	1.525	1.537	1.566
Δ_f_$H_{298}^{0}$, kJ/mol (calcd and exp) [34]	468 calcd	436 calcd 427 exp	490 calcd.

## Data Availability

Not applicable.

## Supplementary Materials

The following are available online at https://www.mdpi.com/article/10.3390/ma15031119/s1: Figures S1–S21: NMR spectra of compounds **2g**, **3g**, **4g**, **3e–3j**, **5e–5h**; Table S1: Crystal data and structure refinement parameters for complex **6**; Table S2: Selected bond distances [Å] for complex **6**.
