# Supplementary material for: Energetic Materials Based on N-substituted 4(5)-nitro-1,2,3-triazoles
Source: Materials (Basel). 2022 Jan 31;15(3):1119. doi: 10.3390/ma15031119 (PMC8838066; doi:10.3390/ma15031119)
Supplement: Supplementary file 1 [file materials-15-01119-s001.zip › materials-1514491-supplementary.pdf]

materials

Supplementary Materials

Energetic Materials Based on N-Substituted 4(5)-Nitro-1,2,3-Triazoles

Gennady. T. Sukhanov [1], Yulia V. Filippova [1,*], Yuri V. Gatilov [2], Anna G. Sukhanova [1], Irina A. Krupnova [1], Konstantin K. Bosov [1], Ekaterina V. Pivovarova [1] and Vyacheslav I. Krasnov [2]

[1] Laboratory for Chemistry and Technology of High-Energy Azoles, Institute for Problems of Chemical and Energetic Technologies, Siberian Branch of the Russian Academy of Sciences (IPCET SB RAS), 659322 Biysk, Russia; suhanovlab7@mail.ru (G.T.S.), nika7_anna@mail.ru (A.G.S.), irinka-krupnova@mail.ru (I.A.K.), kosmos070@gmail.com (K.K.B.), pivovarova.ekaterina@inbox.ru (E.V.P.)

[2] Department of Chemistry, Novosibirsk Institute of Organic Chemistry, Siberian Branch of the Russian Academy of Sciences, 630090 Novosibirsk, Russia; gatilov@nioch.nsc.ru (Y.V.G.), krasnov@nioch.nsc.ru (V.I.K.)

* Correspondence: filippova-yulia@mail.ru; Tel.: +7-3854-30-19-76

1. NMR spectra

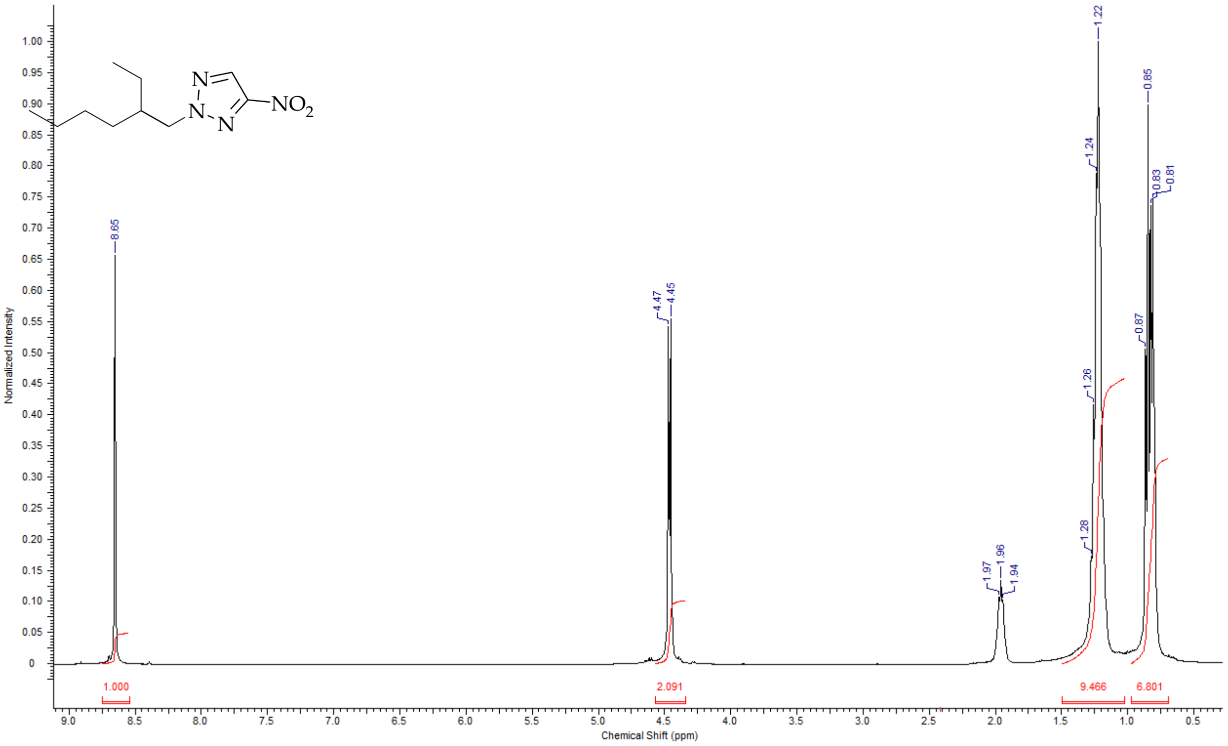

Figure S1. ^{1}H NMR spectrum of 2-ethylhexyl-5-nitro-1.2.3-triazole **3h** in DMSO-d6.

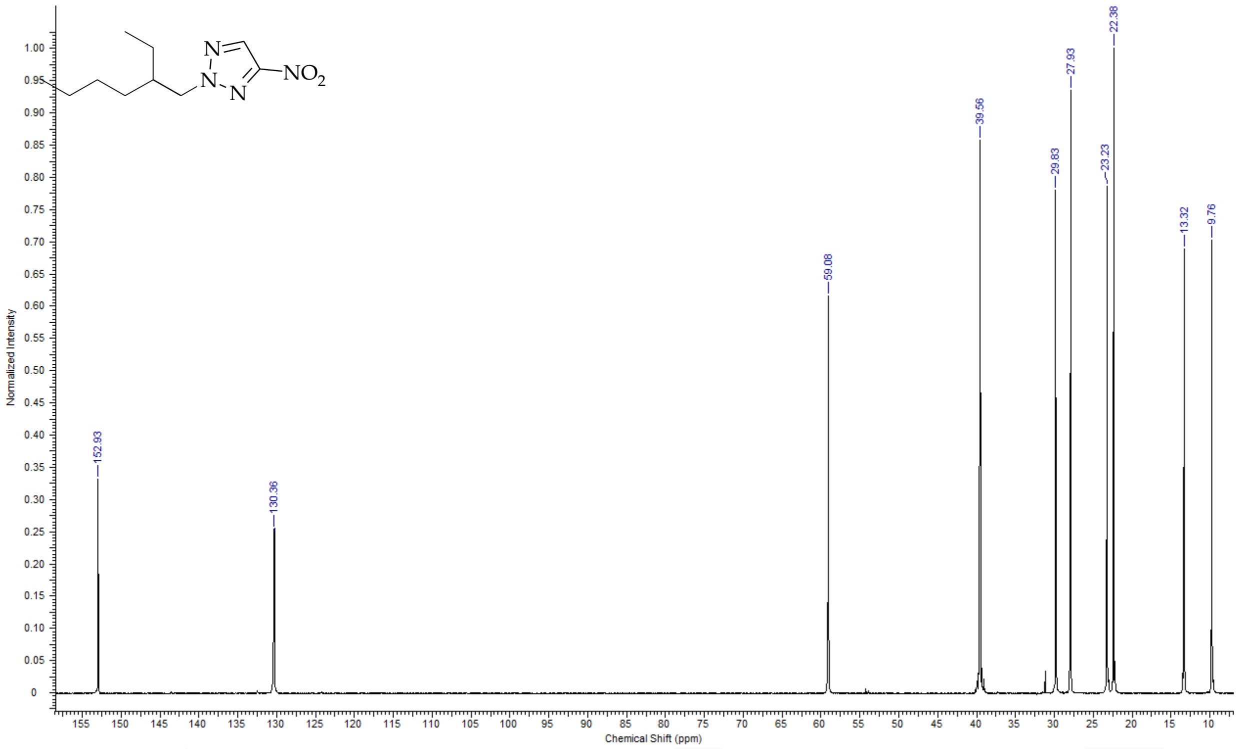

Figure S2. ^{13}C NMR spectrum of 2-ethylhexyl-5-nitro-1.2.3-triazole **3h** in DMSO-d6.

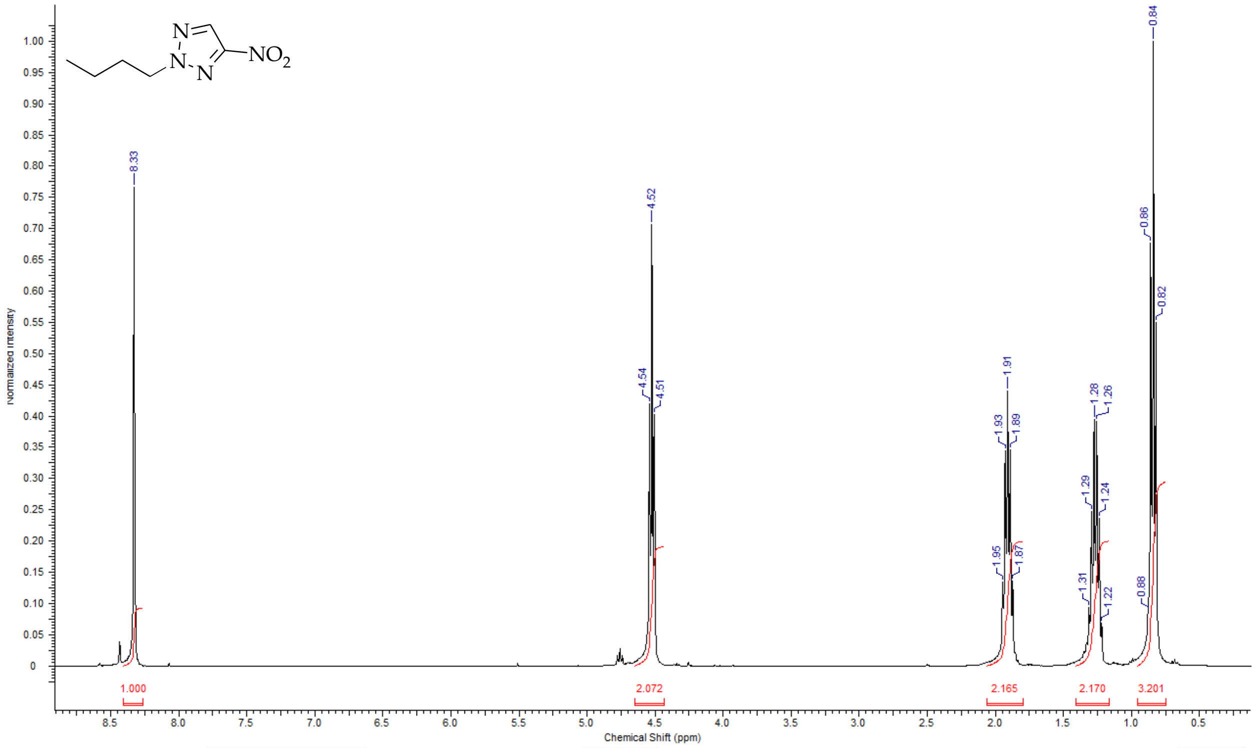

Figure S3. ^{1}H NMR spectrum of 2-n-butyl-4-nitro-1.2.3-triazole **3e** in DMSO-d6.

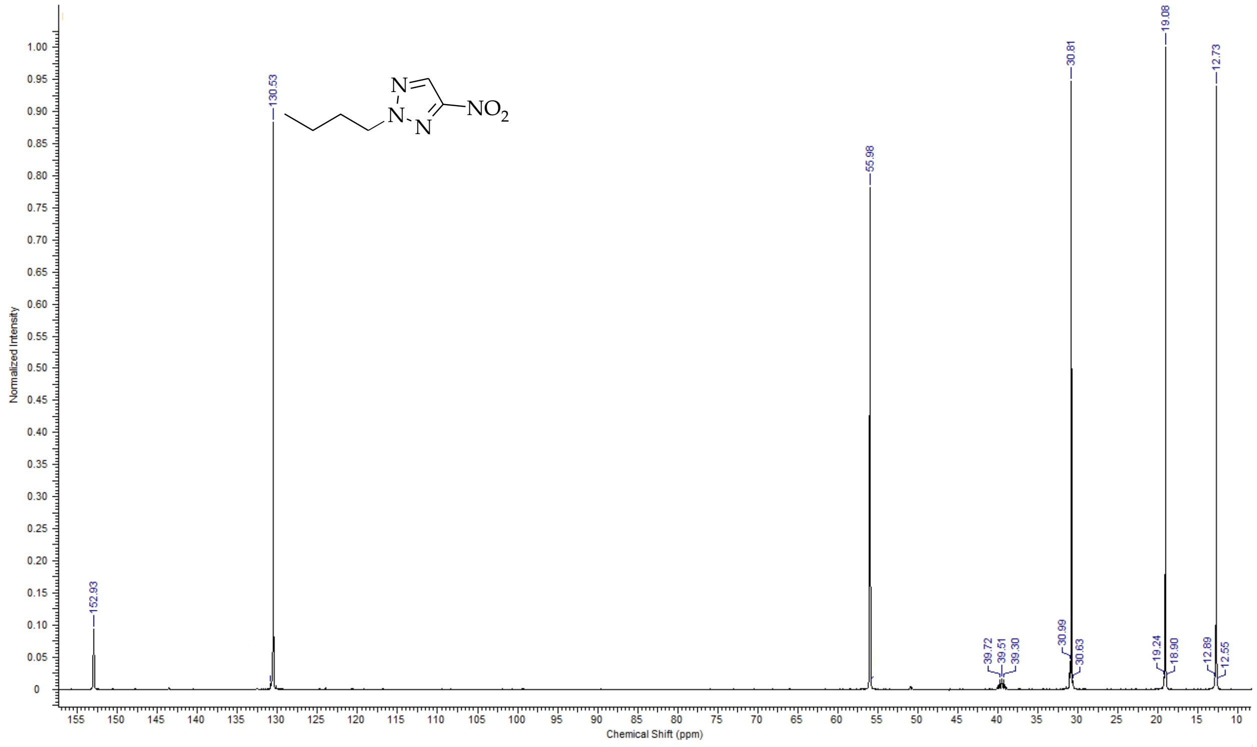

Figure S4. ^{13}C NMR spectrum of 2-n-butyl-4-nitro-1.2.3-triazole **3e** in DMSO-d6.

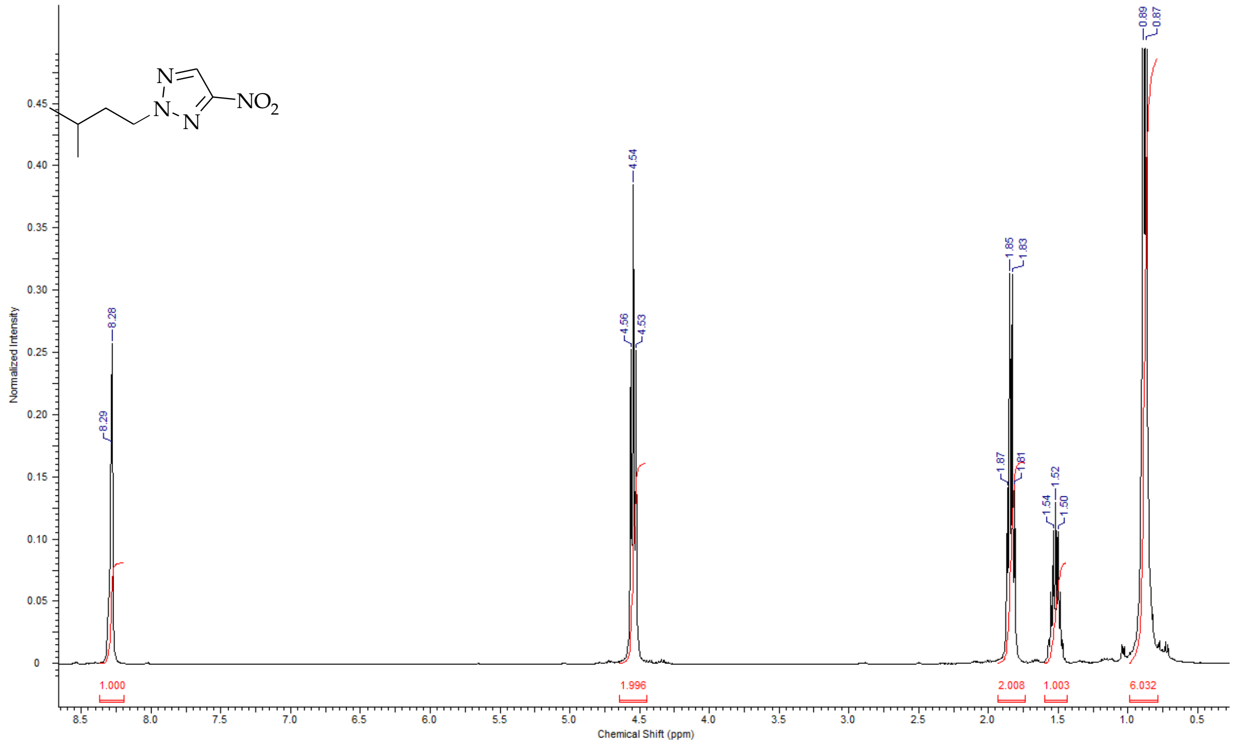

Figure S5. ^{1}H NMR spectrum of 2-i-amyl-5-nitro-1.2.3-triazole **3f** in DMSO-d6.

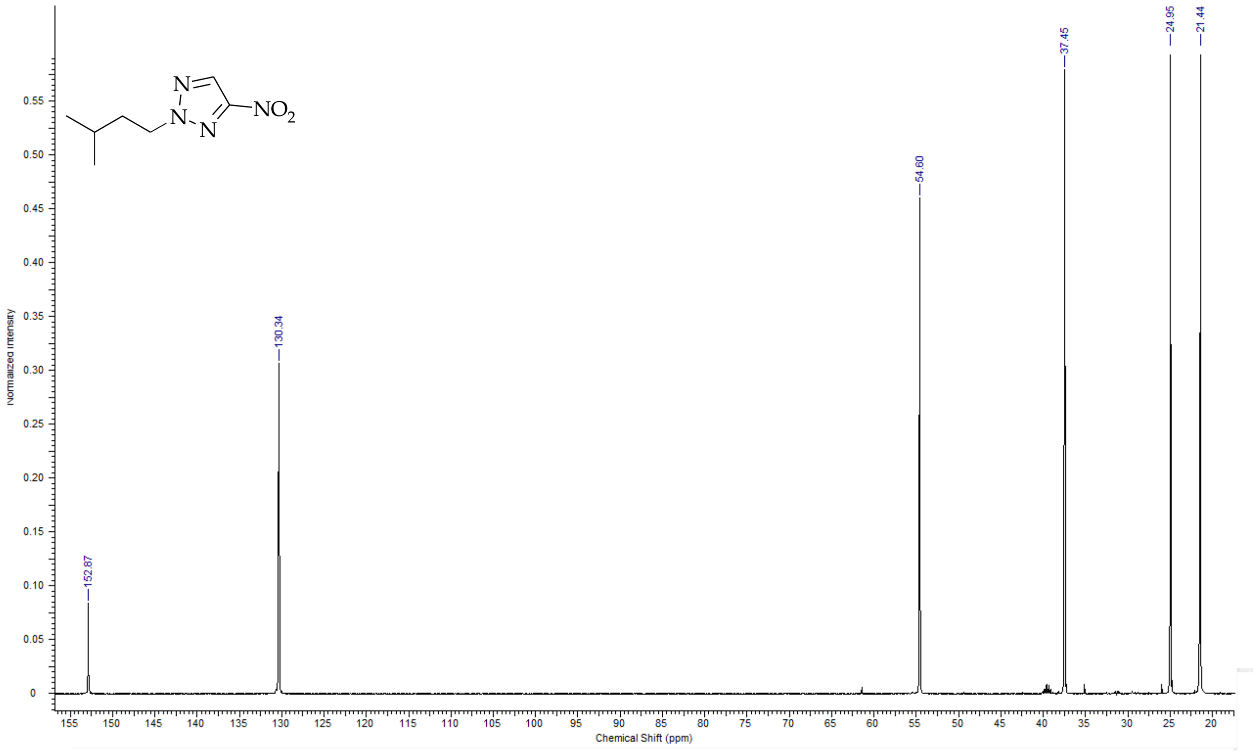

Figure S6. ^{13}C NMR spectrum of 2-i-amyl-5-nitro-1.2.3-triazole **3f** in DMSO-d6.

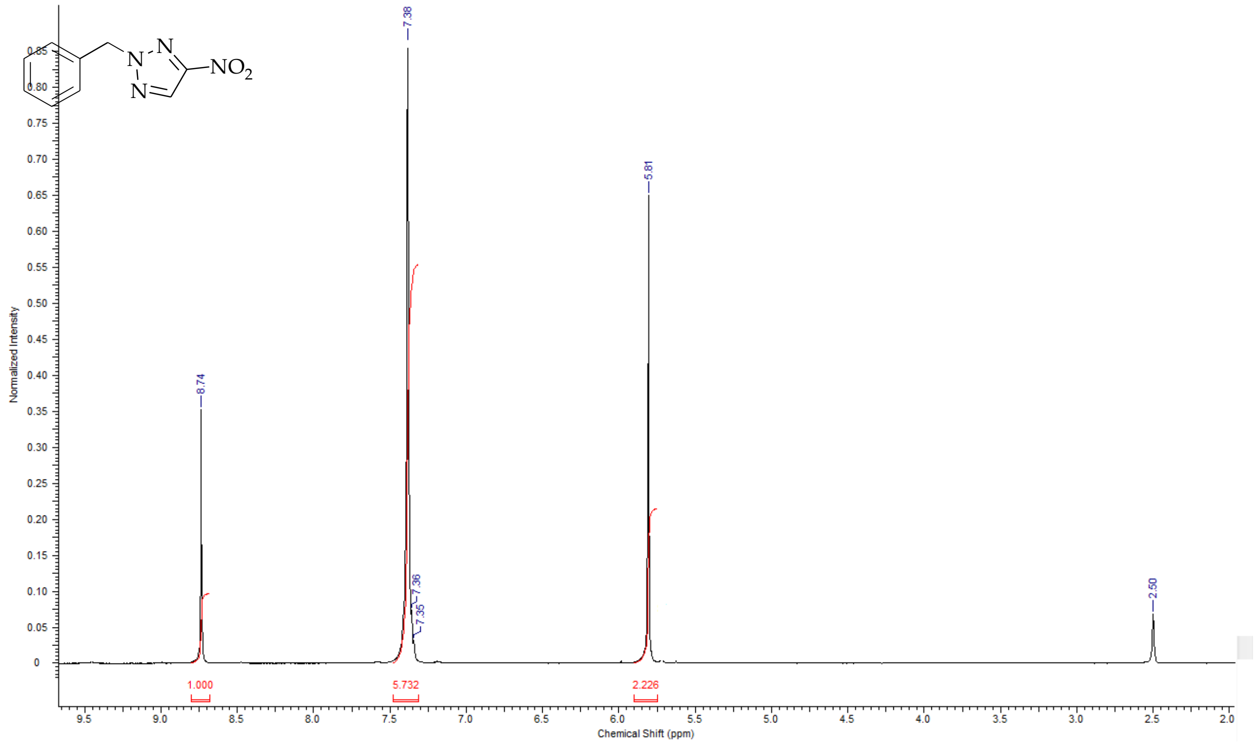

Figure S7. ^{1}H NMR spectrum of 2-benzyl-4-nitro-1.2.3-triazole **3g** in DMSO-d6.

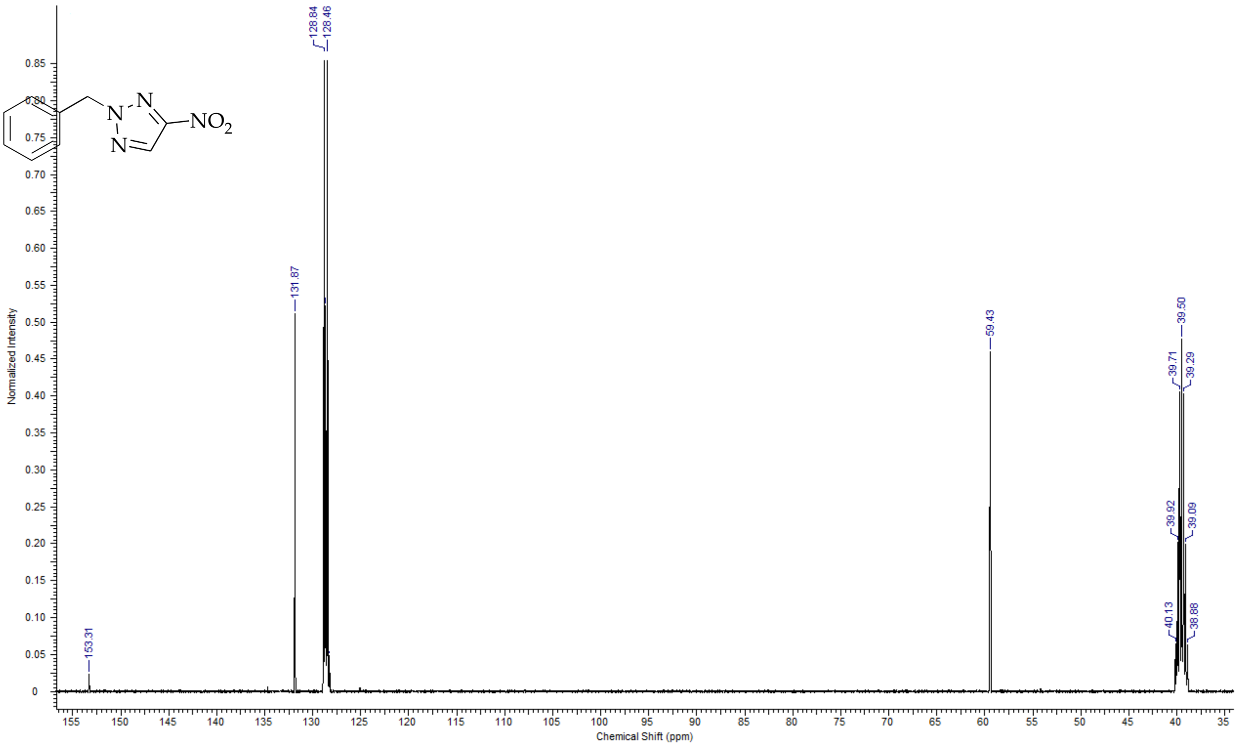

Figure S8. ^{13}C NMR spectrum of 2-benzyl-4-nitro-1.2.3-triazole **3g** in DMSO-d6.

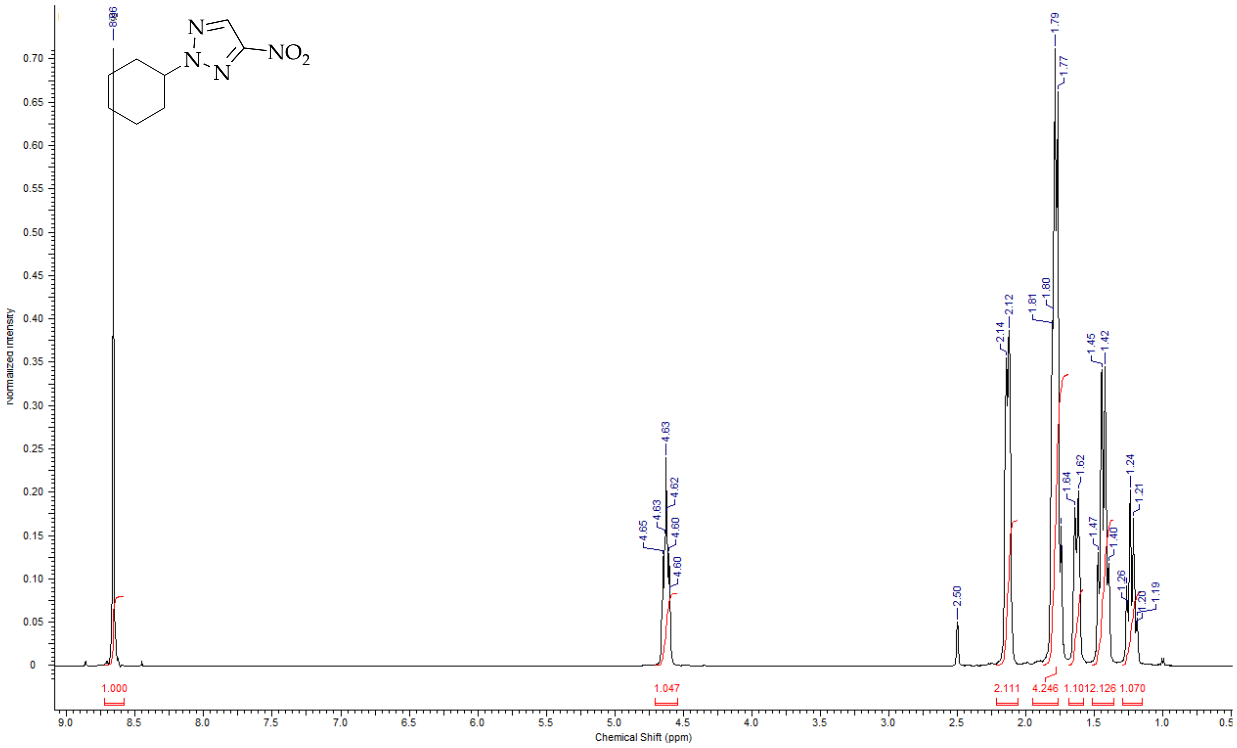

Figure S9. ^{1}H NMR spectrum of 2-cyclohexyl-4-nitro-1.2.3-triazole **3j** in DMSO-d6.

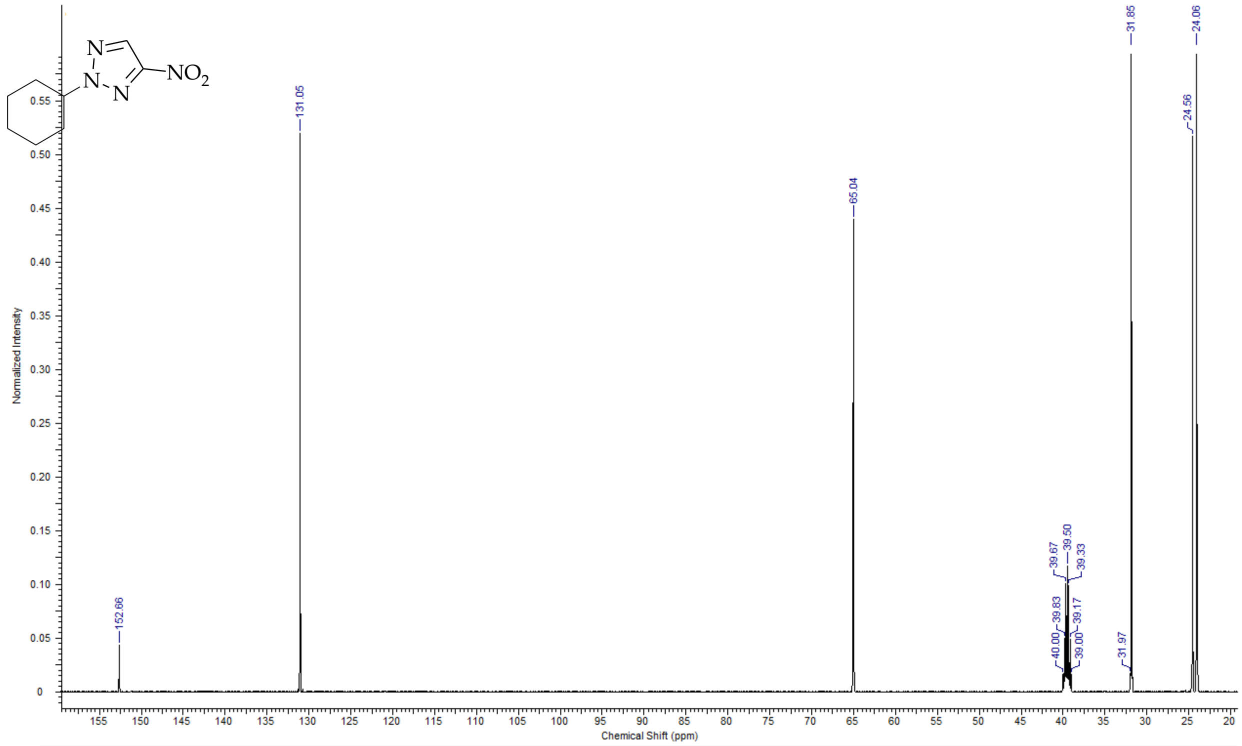

Figure S10. ^{13}C NMR spectrum of 2-cyclohexyl-4-nitro-1.2.3-triazole **3j** in DMSO-d6.

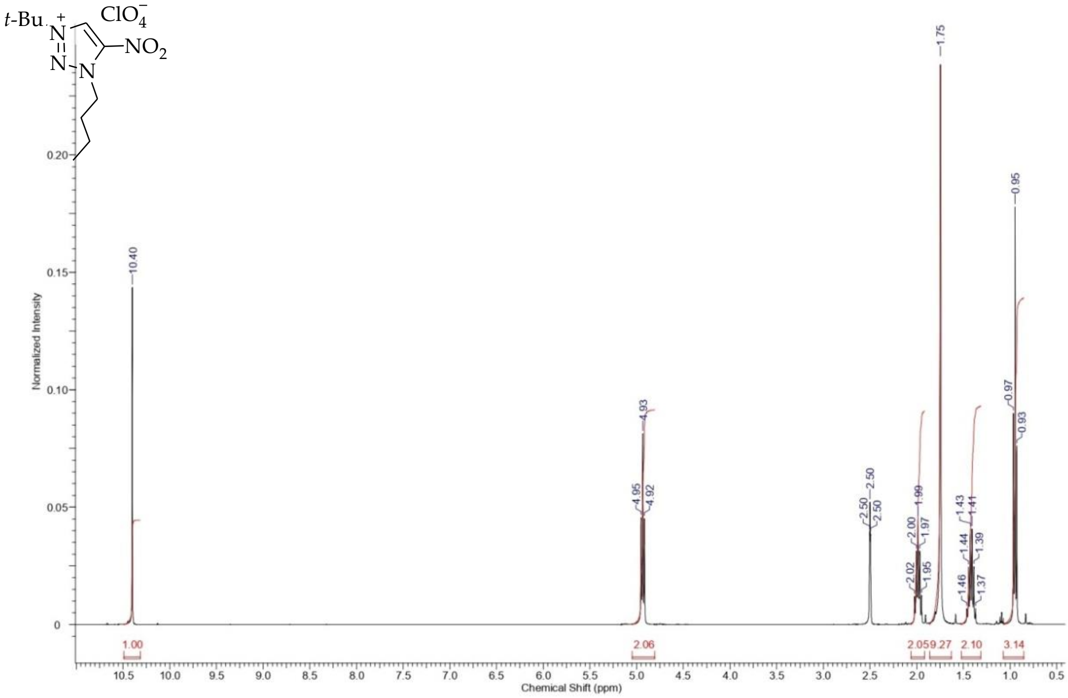

Figure S11. ^{1}H NMR spectrum of 1-tert-butyl-3-n-butyl-4-nitro-1.2.3-triazolium perchlorate **5e** in DMSO-d6.

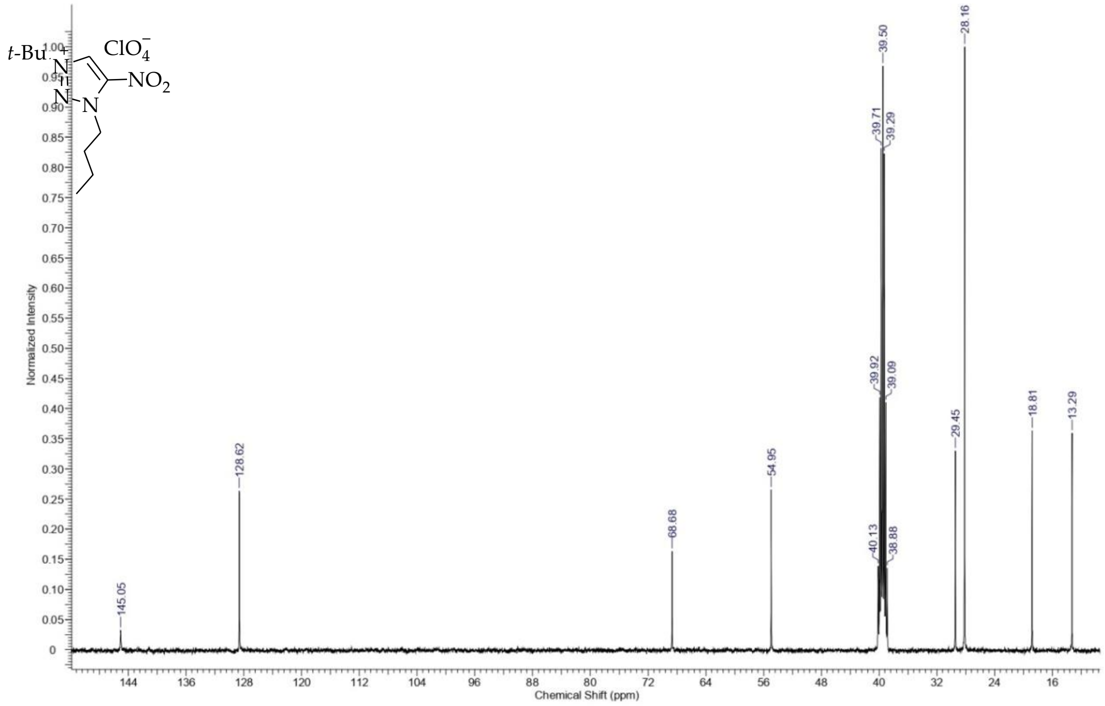

Figure S12. [13] C NMR spectrum of 1-tert-butyl-3-n-butyl-4-nitro-1.2.3-triazolium perchlorate **5e** in DMSO-d6.

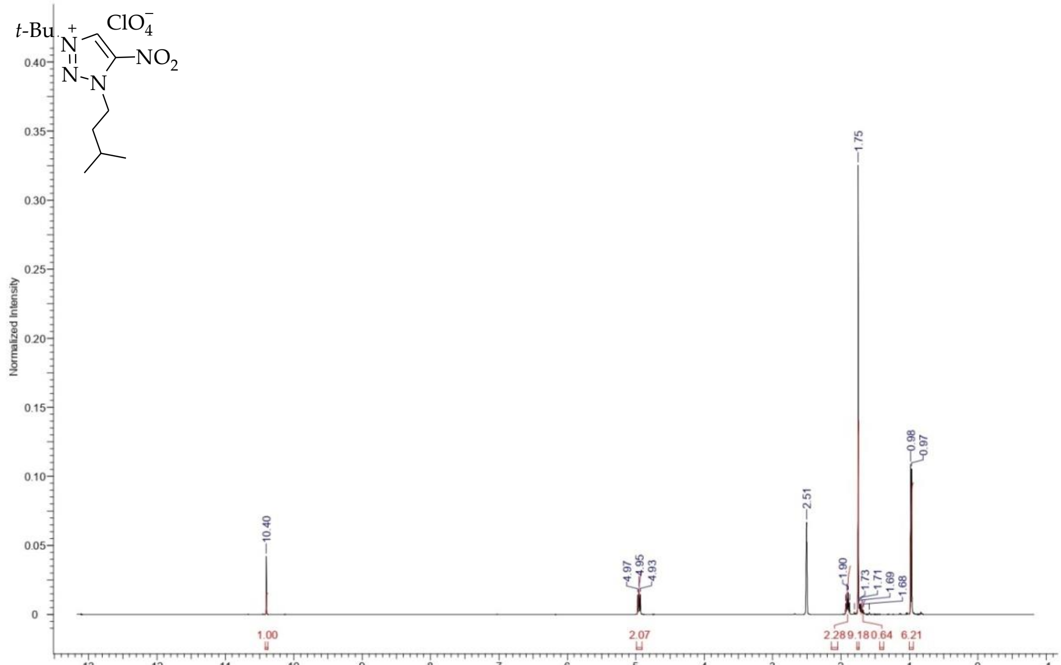

Figure S13. ^{1}H NMR spectrum of 1-tert-butyl-3-i-amyl-4-nitro-1.2.3-triazolium perchlorate **5f** in DMSO-d6.

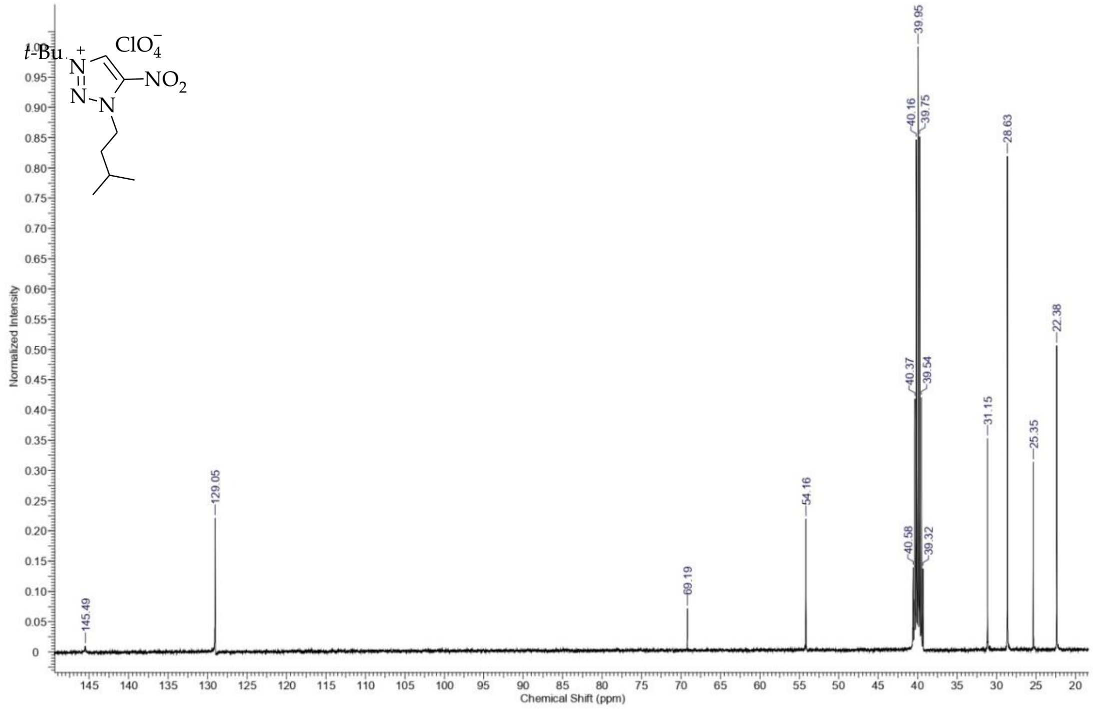

Figure S14. ^{13}C NMR spectrum of 1-tert-butyl-3-i-amyl-4-nitro-1.2.3-triazolium perchlorate **5f** in DMSO-d6.

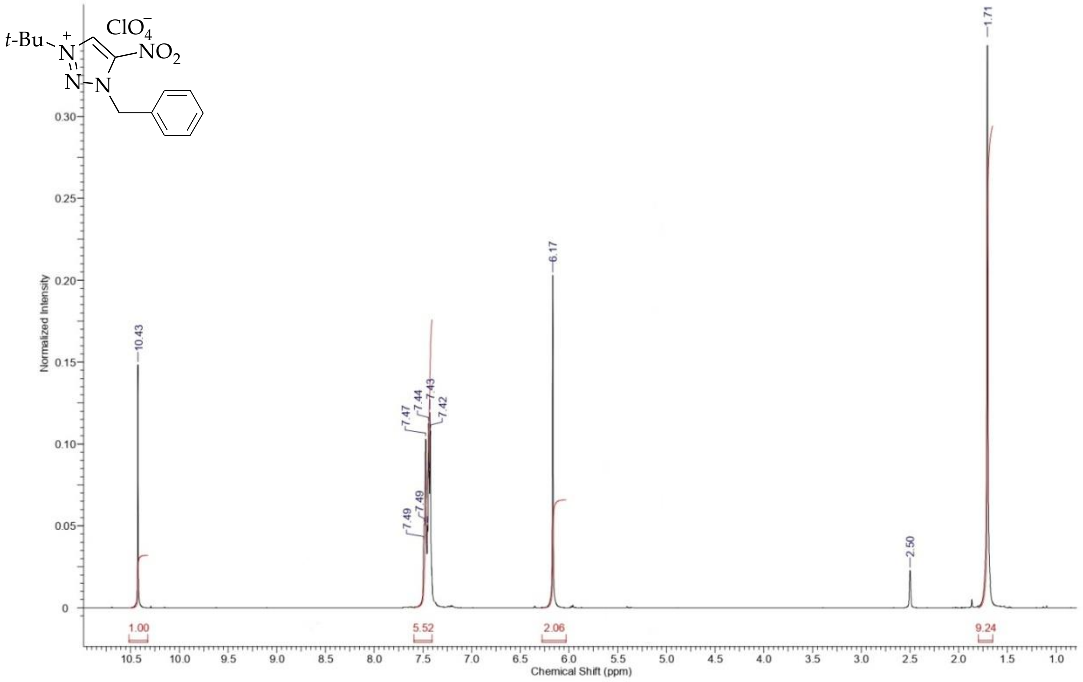

Figure S15. ^{1}H NMR spectrum of 1-tert-butyl-3-benzyl-4-nitro-1.2.3-triazolium perchlorate **5g** in DMSO-d6.

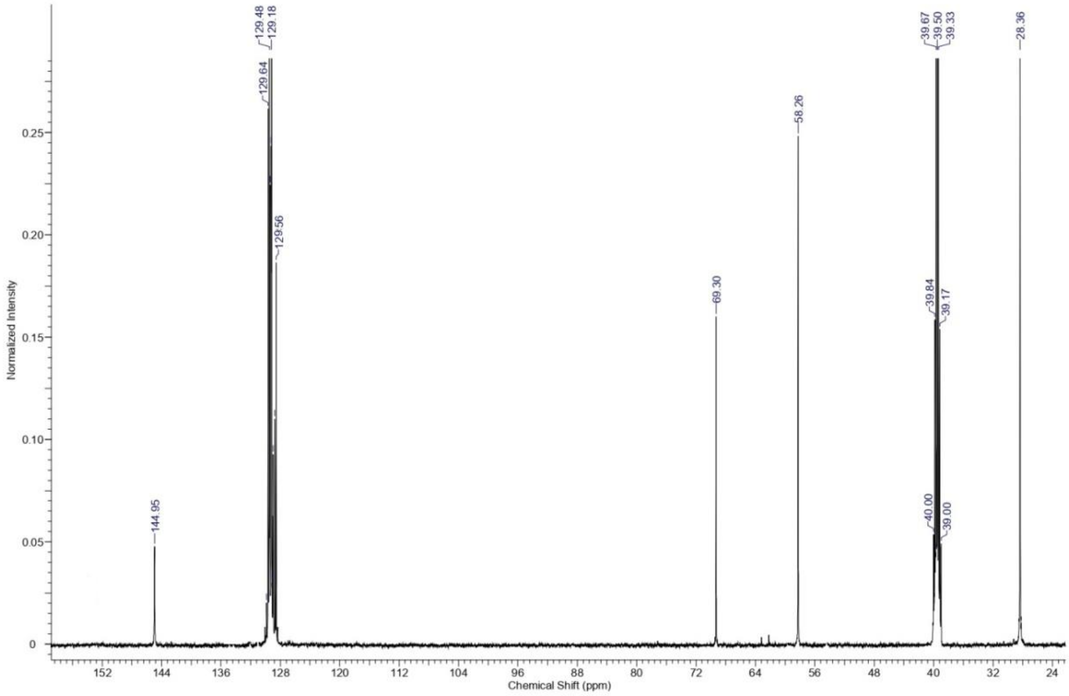

Figure S16. ^{13}C NMR spectrum of 1-tert-butyl-3-benzyl-4-nitro-1.2.3-triazolium perchlorate **5g** in DMSO-d6.

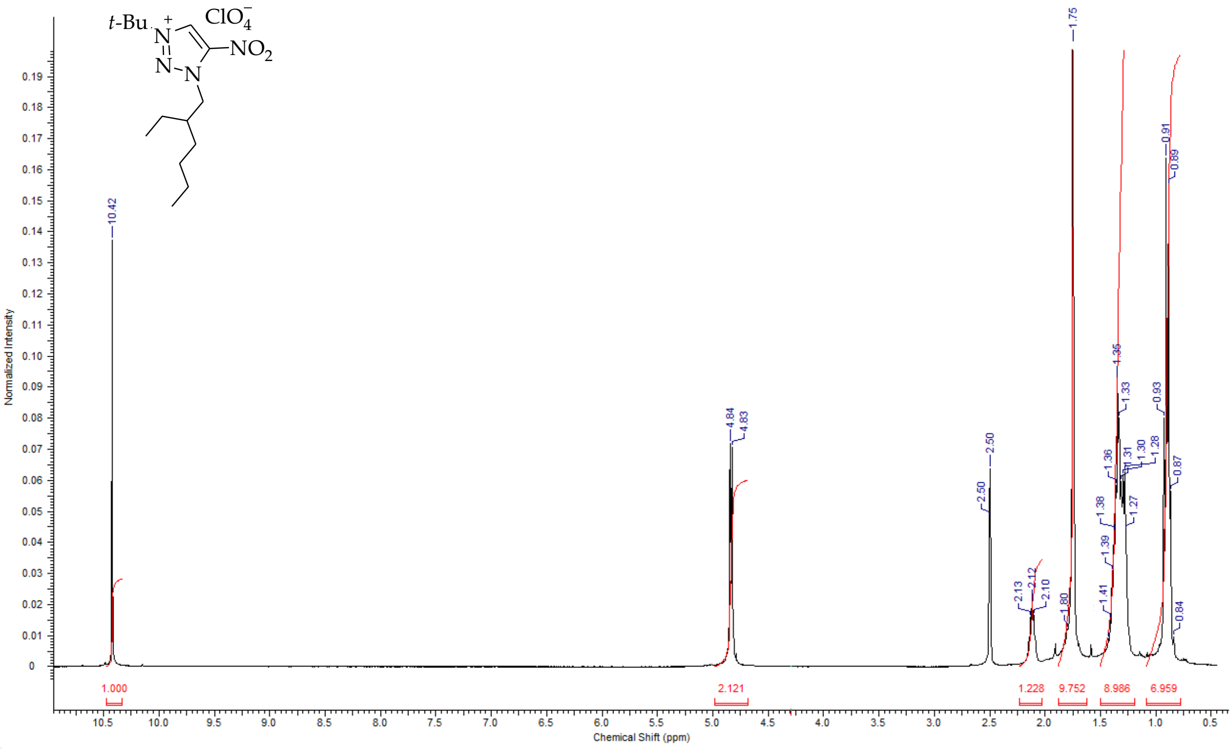

Figure S17. ^{1}H NMR spectrum of 1-tert-butyl-3-ethylhexyl-4-nitro-1.2.3-triazolium perchlorate **5h** in DMSO-d6.

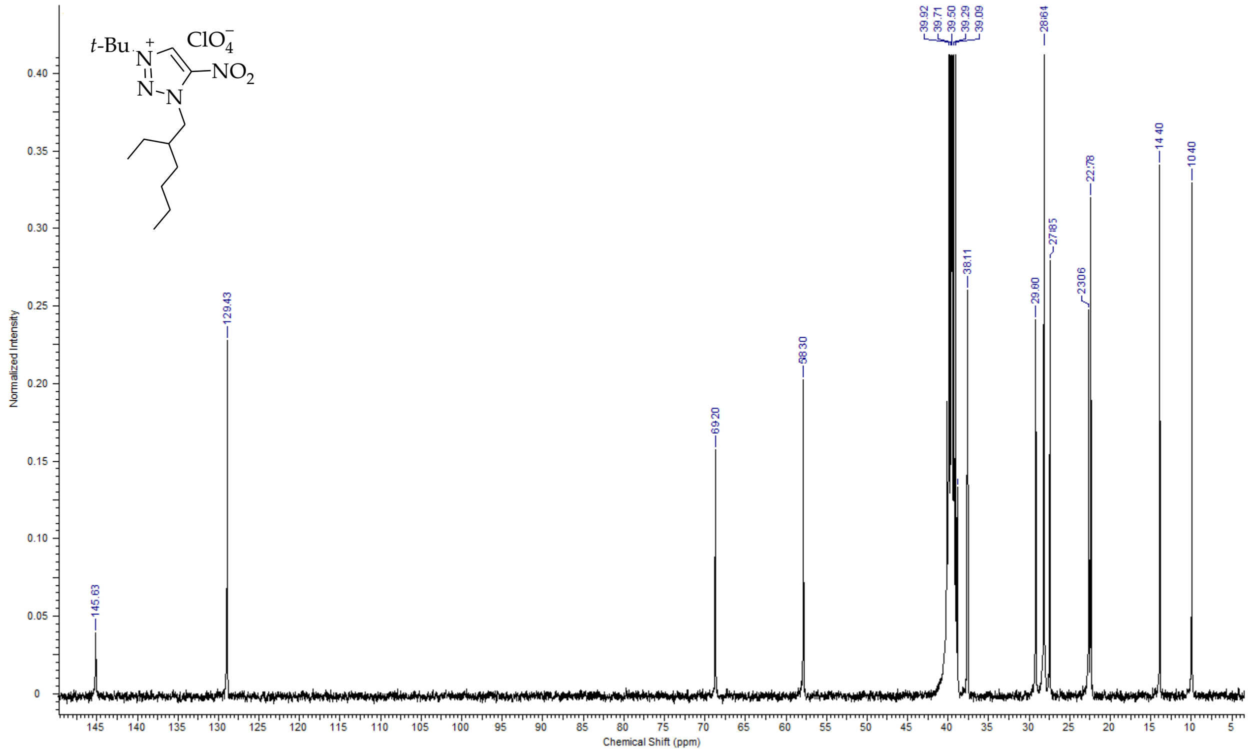

Figure S18. ^{13}C NMR spectrum of 1-tert-butyl-3-ethylhexyl-4-nitro-1.2.3-triazolium perchlorate **5h** in DMSO-d6.

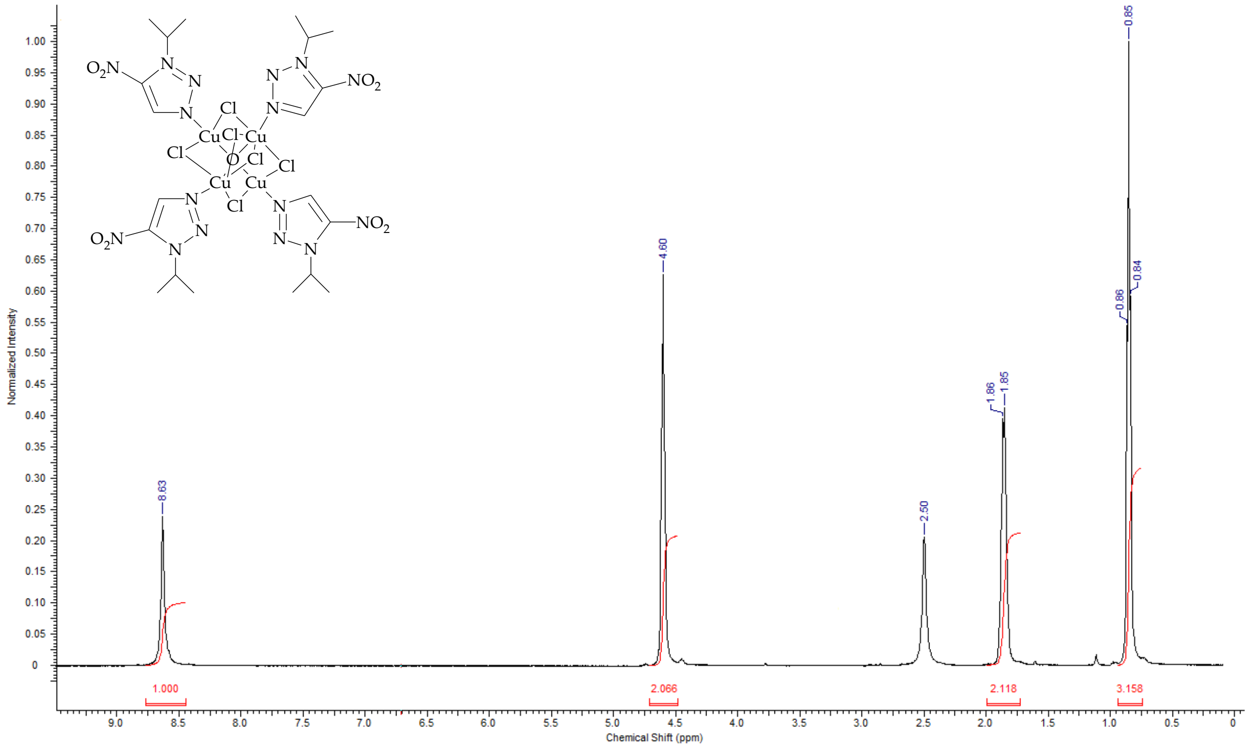

Figure S19. ^{1}H NMR spectrum of complex **6** in DMSO-d6.

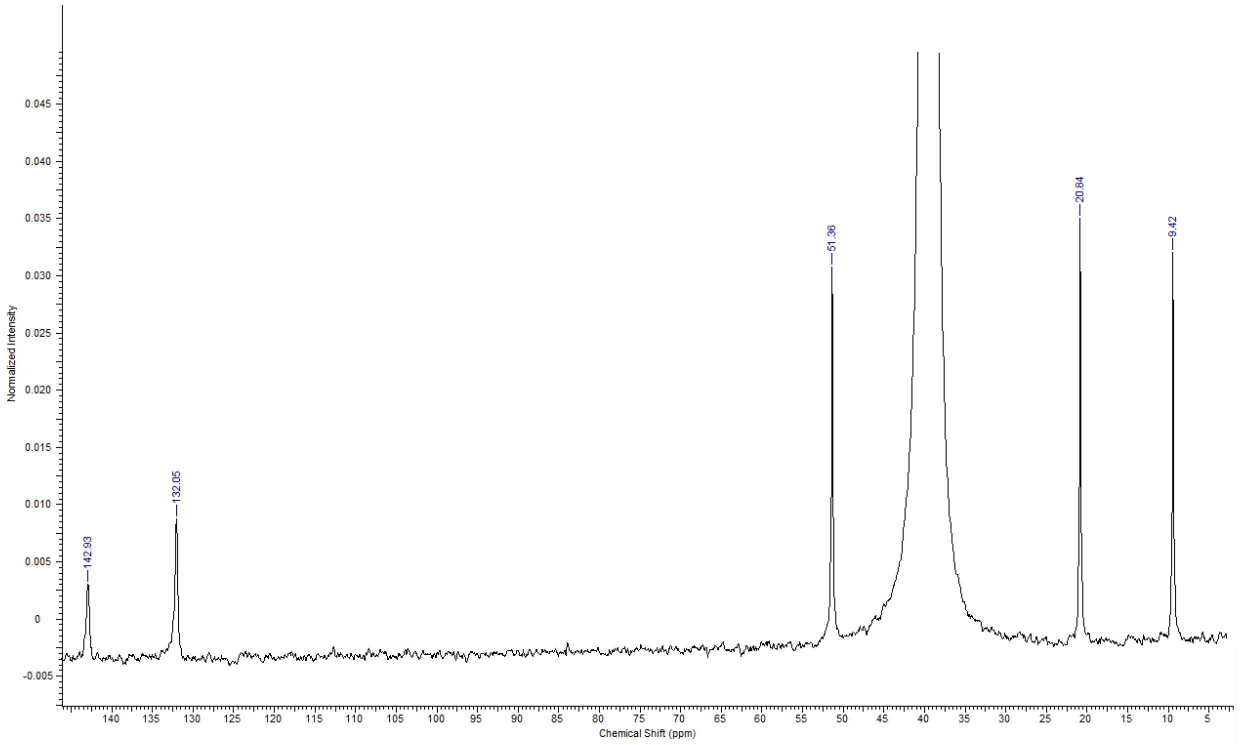

Figure S20. ^{13}C NMR spectrum of complex **6** in DMSO-d6.

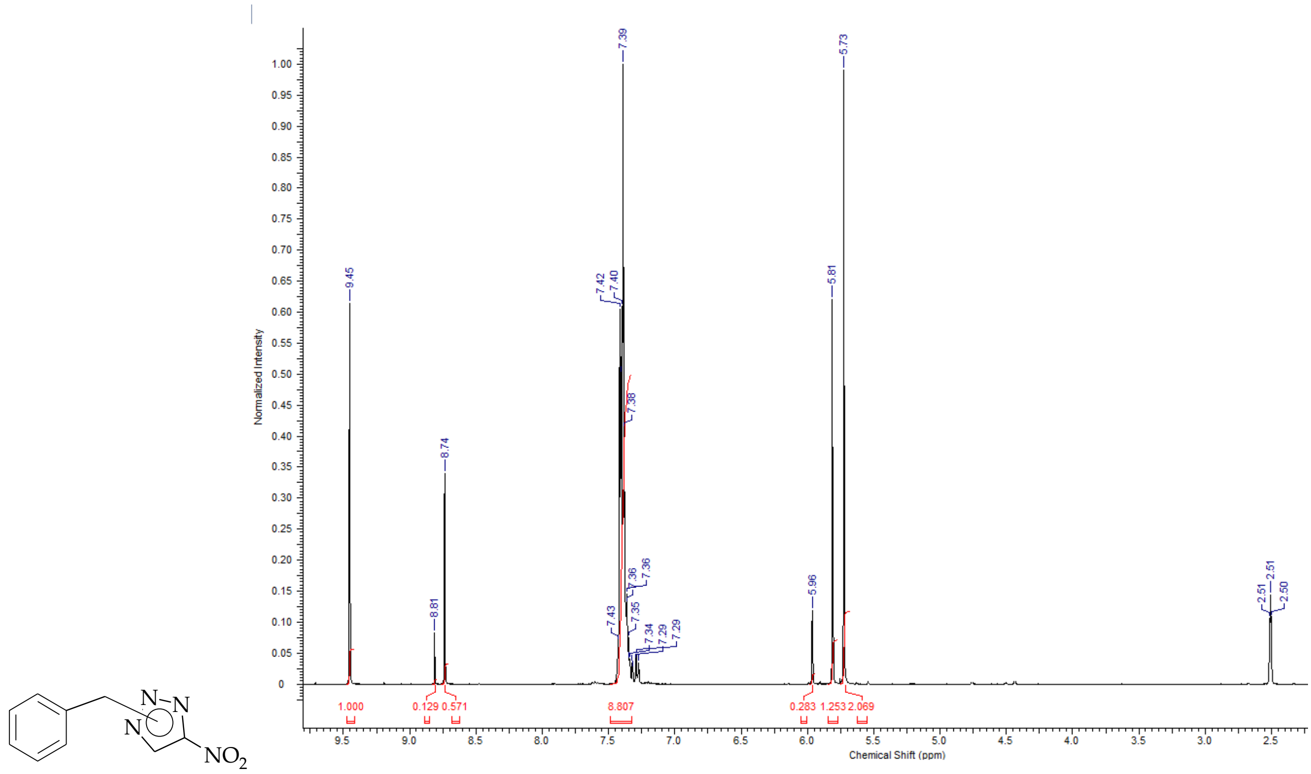

Figure S21. ^{1}H NMR spectrum of mixture N-benzyl-4-nitro-1.2.3-triazoles **2–4g** in DMSO-d6.

2. X-ray Crystallography Study

Single crystal X-ray diffraction intensity data of were collected at 296(2) K using a Bruker APEX-II CCD diffractometer equipped with graphite monochromated MoKα radiation (λ = 0.71073 Å). Data reduction was carried out using the program Bruker SAINT and an empirical absorption correction was applied with Bruker SADABS program based on multi-scan method. The structure of the complex was solved by direct method (SHELXT-18) and refined by the full-matrix least-square technique (SHELXL-18) with anisotropic thermal parameters. All hydrogen atoms were refined isotropically in riding positions. The summary of crystal data and relevant structure refinement parameters are given in Table S1. Selected Bond Distances are given in Table S2. CCDC 2119451 contain the supplementary crystallographic data.

Table S1. Crystal Data and Structure Refinement Parameters for Complex **6**.

**formula**	$C_{20}H_{32}Cl_6Cu_4N_{16}O_9$
**formula weight**	1107.47
**crystal system**	triclinic
**space group**	P-1
**a, b, c (Å)**	12.2006(4), 12.4787(5), 15.6707(6)
**α, β, γ (deg)**	110.386(1), 109.206(1), 93.233(1)
**volume (Å^3)**	2072.15(14)
**Z/density (calc.) (Mg/m^3)**	2/1.775
**absorption coefficient (mm^{-1})**	2.475
**crystal size (mm^3)**	0.19 × 0.31 × 0.38
**θ range for data collection (deg)**	2.3, 30.2
**reflections collected/unique**	72174/12239
**completeness to θ (%)**	99.4
**max. and min transmission**	0.6948, 0.5698
**data/restraints/parameters**	12239/0/500
**goodness-of-fit**	1.01
**final R indices [I > 2σ(I)]**	R=0.0333, wR_2=0.0979
**R indices (all data)**	R=0.0468, wR_2=0.1151
**largest diff. peak and hole (e/Å^3)**	−0.58, 0.78

Table S2. Selected Bond Distances [Å] for Complex **6**.

**Bonds**	**Distances**	**Bonds**	**Distances**	**Bonds**	**Distances**
Cu1-O1	1.9038(14)	Cu1-N1	1.972(2)	Cu1-Cl3	2.3763(7)
Cu1-Cl1	2.3779(6)	Cu1-Cl2	2.4474(6)	-	-
Cu2-O1	1.8958(14)	Cu2-N10	1.972(2)	Cu2-Cl5	2.3622(7)
Cu2-Cl1	2.4256(6)	Cu2-Cl4	2.4354(6)	-	-
Cu3-O1	1.8997(14)	Cu3-N19	1.9793(19)	Cu3-Cl2	2.3575(7)
Cu3-Cl4	2.4250(6)	Cu3-Cl6	2.4577(7)	-	-
Cu4-O1	1.8922(14)	Cu4-N28	1.974(2)	Cu4-Cl6	2.3384(6)
Cu4-Cl3	2.3786(7)	Cu4-Cl5	2.5233(7)	-	-
